# Deliberative and Paternalistic Interaction Styles for Conversational Agents in Digital Health: Procedure and Validation Through a Web-Based Experiment

**DOI:** 10.2196/22919

**Published:** 2021-01-29

**Authors:** Theresa Schachner, Christoph Gross, Andrea Hasl, Florian v Wangenheim, Tobias Kowatsch

**Affiliations:** 1 Department of Management, Technology, and Economics Centre for Digital Health Interventions ETH Zurich Zurich Switzerland; 2 Department of Educational Sciences University of Potsdam Potsdam Germany; 3 International Max Planck Research School on the Life Course (LIFE) Berlin Germany; 4 Centre for Digital Health Interventions Institute of Technology Management University of St Gallen St. Gallen Switzerland

**Keywords:** conversational agents, chatbots, human-computer interaction, physician-patient relationship, interaction styles, deliberative interaction, paternalistic interaction, digital health, chronic conditions, COPD

## Abstract

**Background:**

Recent years have witnessed a constant increase in the number of people with chronic conditions requiring ongoing medical support in their everyday lives. However, global health systems are not adequately equipped for this extraordinarily time-consuming and cost-intensive development. Here, conversational agents (CAs) can offer easily scalable and ubiquitous support. Moreover, different aspects of CAs have not yet been sufficiently investigated to fully exploit their potential. One such trait is the interaction style between patients and CAs. In human-to-human settings, the interaction style is an imperative part of the interaction between patients and physicians. Patient-physician interaction is recognized as a critical success factor for patient satisfaction, treatment adherence, and subsequent treatment outcomes. However, so far, it remains effectively unknown how different interaction styles can be implemented into CA interactions and whether these styles are recognizable by users.

**Objective:**

The objective of this study was to develop an approach to reproducibly induce 2 specific interaction styles into CA-patient dialogs and subsequently test and validate them in a chronic health care context.

**Methods:**

On the basis of the Roter Interaction Analysis System and iterative evaluations by scientific experts and medical health care professionals, we identified 10 communication components that characterize the 2 developed interaction styles: deliberative and paternalistic interaction styles. These communication components were used to develop 2 CA variations, each representing one of the 2 interaction styles. We assessed them in a web-based between-subject experiment. The participants were asked to put themselves in the position of a patient with chronic obstructive pulmonary disease. These participants were randomly assigned to interact with one of the 2 CAs and subsequently asked to identify the respective interaction style. Chi-square test was used to assess the correct identification of the CA-patient interaction style.

**Results:**

A total of 88 individuals (42/88, 48% female; mean age 31.5 years, SD 10.1 years) fulfilled the inclusion criteria and participated in the web-based experiment. The participants in both the paternalistic and deliberative conditions correctly identified the underlying interaction styles of the CAs in more than 80% of the assessments (*X*^2^_1,88_=38.2; *P*<.001; phi coefficient *r*_φ_=0.68). The validation of the procedure was hence successful.

**Conclusions:**

We developed an approach that is tailored for a medical context to induce a paternalistic and deliberative interaction style into a written interaction between a patient and a CA. We successfully tested and validated the procedure in a web-based experiment involving 88 participants. Future research should implement and test this approach among actual patients with chronic diseases and compare the results in different medical conditions. This approach can further be used as a starting point to develop dynamic CAs that adapt their interaction styles to their users.

## Introduction

### Background

The interaction between patients and physicians is recognized as a critical success factor for treatment satisfaction, adherence, and subsequent treatment outcomes [1,2]. Its importance has been shown in face-to-face encounters between patients and physicians and in distance therapy via, for example, phone or internet [3,4]. A previous study [5] has differentiated between 4 distinct interaction styles between patients and physicians: paternalistic, informative, interpretative, and deliberative interaction styles. The paternalistic interaction style is characterized by physicians acting as “guardian” [5] of patients and paternally making decisions grounded in the assumption of objective and shared values between them and their patients. Although applying the informative interaction style, physicians act as “competent technical expert” [5], passing on information to their patients who ultimately have decision control based on their personal values. Within the interpretative interaction style, patients are confronted with medical information but are unsure about how to deal with them. Here, physicians act as a “counsellor or advisor” [5], helping patients to make their decision based on better self-understanding. Finally, physicians act as a “teacher or friend” [5] while engaging in a deliberative interaction style. They present medical information, promote particular health-related values, and conjointly discuss the best way forward together with their patients. This style can also be described as shared decision making and is advocated by contemporary medical research [6]. Especially while addressing patient autonomy in chronic care, a deliberative patient-physician interaction is thought to activate patients’ intrinsic values and goals better than any other interaction style and is, therefore, believed to be preferable [7].

However, such shared decision making is not always possible or even desired by patients. For example, patients seem to prefer paternalistic interactions in acute care conditions, especially when they have low health literacy or are emotionally overburdened by the situation [8]. Recent works have also shown that older people [9,10], men [9,10], less educated patients [10], patients with physical problems, and patients with severe exacerbation of their condition [11,12] prefer paternalistic interactions with their physicians. In addition, personal preferences for a preferred interaction style can change over time [13].

The personalization of physician-patient interactions thus seems to be appropriate while aiming at optimally adapting to the needs of the patients. This holds special importance in the context of chronic diseases, where patients have to deal with their condition for a prolonged period, experience functional limitations, require ongoing medical support, and often undergo several exacerbations of their condition [14-16]. In addition to continuing treatment and medical supervision of such chronic conditions, which pose an increasingly higher risk to the world population and are an enormous financial burden for global health care systems [17], it is important to engage in active disease management. Effective disease management includes educational measures, behavior modification, and psychological support [18] and can minimize overall harm and long-term effects of chronic conditions as well as confine exacerbation risks [19]. However, disease management is labor intensive, time-consuming, and costly for trained medical staff [20-23].

Against this background, emerging digital health tools such as conversational agents (CAs) offer hope in supporting patients’ self-management of their conditions. CAs are software programs that imitate natural interactions with human users by engaging in a human-like text-based and/or voice-enabled dialog [24,25]. Recent research has shown the ability of CAs to positively affect patient satisfaction [26], therapeutic alliance [27,28], and health-related outcomes [29,30]. Moreover, an overall acceptability has been established in various populations [31]. As scalable and ubiquitous digital tools, CAs facilitate personalized disease management outside the traditional health care system. Relevant aspects for developing personalized CAs, such as the required level of anthropomorphic appearance [32] or necessary design features [33], have been extensively investigated. However, to date, there has been no investigation of different interaction styles between patients and health care professionals where CAs play the role of medical experts. As mentioned above, these several forms of patient-physician interaction styles exert a significant effect on treatment success. We thus assume that appropriate interaction styles are also indispensable for patient-CA interactions. As a first step for developing personalized CAs that can adapt to the needs of the patient at hand and even adjust to changing individual preferences over time, it is essential to first develop and validate a systematic approach to develop and induce several interaction styles into patient-CA interactions.

### Objectives

To this end, the objectives of this study were to (1) create and present a systematic approach to develop and induce specific interaction styles specifically for health care CAs and (2) validate whether individuals can correctly differentiate between the induced interaction styles. Our overarching research question for this paper is as follows: are humans capable of correctly identifying and labeling either an induced deliberative or paternalistic interaction style while interacting with a health care CA?

## Methods

### Development of CAs

In a 3-step process, we have developed a comprehensive mechanism to induce a deliberative and paternalistic interaction style into a CA interaction. These 3 steps are (1) development of the 2 interaction styles in the form of interaction items and a corresponding measurement scale, (2) scripting of CAs based on developed interaction styles, and (3) validation of the developed CAs in a web-based experiment.

We decided to induce deliberative and paternalistic interaction styles [5] as they are the 2 endpoints of a patient’s autonomy spectrum [34]. Here, by means of education, patient autonomy and mutual trust increase from the paternalistic to the deliberative interaction style. Patient autonomy is understood as the ability to accept one’s treatment preferences or to change to higher-order preferences through deliberation [34].

We first compiled an initial list of 28 communication components (Multimedia Appendix 1 [1,35-39]) that we adopted from the communication behaviors established by the Roter Interaction Analysis System (RIAS) [35]. The RIAS is a widely applied communication coding scheme for medical dialogs and assigns recorded verbal utterances to distinct categories [35]. Communication components combine communication clusters with communication categories to specify an explicit utterance in a dialog. To illustrate, consider the communication component “Therapeutic regimen_closed-ended question.” It combines the communication cluster “Therapeutic regimen” with the communication category “closed-ended question” and denotes a remark by the physician about a patient’s therapeutic regimen, posed as a closed-ended question. All communication components are described from the physician’s point of view. On the basis of the related scientific literature [1,36,40-42], we assigned frequency levels (high or low) that are characteristic of a deliberative and paternalistic interaction style to each of the 28 communication components in the next step. For example, we allocated a high-frequency level of the communication component “Medical condition_Open-ended question” to the deliberative interaction style and a low frequency to the paternalistic one. Three health care practitioners of a European University Hospital have close experience with and exposure to both teaching and various medical communication techniques. They were thus qualified to review and endorse the authors’ work.

To refine our proposed approach and improve its practicability, we aimed at reducing the total number of communication components. First, we excluded 6 communication components with identical frequency assignments for both the deliberative and paternalistic interaction styles, as they did not yield exclusive information on any of the 2 interaction strategies. An example would be “Biomedical information_About medical condition,” where frequency levels were high for both the interaction styles. This initial triage resulted in a total of 22 remaining communication components. On the basis of our theoretical knowledge, we selected a binary choice between including and not including 15 of these components that seemed most relevant to distinguish between paternalistic and deliberative patient-physician-interactions. To triangulate our item selection and minimize the risk of bias, we asked 2 junior medical doctors with work experience less than 5 years since graduation and 2 experienced medical practitioners with more than 20 years of work experience to identify the 15 components they felt were the most relevant to differentiate between the 2 interaction styles. We deliberately chose to incorporate junior physicians because they are trained in the currently advocated deliberative interaction style and senior physicians because they are still familiar with the traditional paternalistic interaction style. The 4 physicians had different medical specializations. This approach allowed us to include both theoretical and practical perspectives while reducing the risk of bias in the item reduction process. To determine the intersecting set of communication components, we calculated the inter-rater reliability (IRR) across all the communication components [43]. Following this approach, we ensured the highest objectivity in the selection process. Consistent with a previous work [43], we chose 80% as the cut-off rate for the inclusion of an item. The evaluation ratings can be found in Multimedia Appendix 2. The final list resulted in 10 communication components, as listed in Table 1 (with example statements detailing the communication components) with their respective frequency measures for the 2 interaction styles. Among the final choices of the 10 communication components, 7 showed an IRR of 100%. Hence, all the 6 raters (ie, the main authors, TS and CG, and the 4 medical practitioners) selected these components to be the most relevant to differentiate between the 2 interaction styles.

**Table 1:** A final list of communication components.

ID	Communication component	Communication frequency		Example statement
		Paternalism	Deliberative	
CC1	Medical condition_open-ended question	Low	High	What can you tell me about the pain? [35]
CC2	Therapeutic regimen_open-ended question	Low	High	How are your symptoms developing since you take the new pills? (developed by authors, adapted from the studies by Ong et al, 1995; Cavaco and Roter, 2010; Roter et al, 1997; and Cegala, 1997 [1,35-37])
CC3	Therapeutic regimen_closed-ended question	High	Low	Did you reduce your cigarette consumption to max. 10 cigarettes/day as discussed? (developed by authors, adapted from the studies by Ong et al, 1995; Cavaco and Roter, 2010; Roter et al, 1997; and Cegala, 1997 [1,35-37])
CC4	Psychosocial Exchange about problems of daily living, issues about social relations, feelings, emotions	Low	High	It is important to talk about your worries regarding your condition (developed by authors, adapted from the studies by Ong et al, 1995; Cavaco and Roter, 2010; Roter et al, 1997; and Cegala, 1997 [1,35-37])
CC5	Emotional Talk_Reassurance/Optimism	Low	High	Your arm will feel better soon, no worries! (developed by authors, adapted from the studies by Ong et al, 1995; Cavaco and Roter, 2010; Roter et al, 1997; and Cegala, 1997 [1,35-37])
CC6	Emotional Talk_Empathy	Low	High	I can see how worried you are from hearing these results of your lung test. (developed by authors, adapted from the studies by Ong et al, 1995; Cavaco and Roter, 2010; Roter et al, 1997; and Cegala, 1997 [1,35-37])
CC7	Emotional Talk_Partnership	Low	High	We’ll get through this together [35]
CC8	Partnering and activation_Asking for patient opinion	Low	High	Do you want to bring your husband to the next session? (developed by authors, adapted from the studies by Ong et al, 1995; Cavaco and Roter, 2010; Roter et al, 1997; and Cegala, 1997 [1,35-37])
CC9	Partnering and activation_Asking for understanding	Low	High	Can you follow all my instructions? (developed by authors, adapted from the studies by Ong et al, 1995; Cavaco and Roter, 2010; Roter et al, 1997; and Cegala, 1997 [1,35-37])
CC10	Partnering and activation_Paraphrase and interpretation	Low	High	Ok, let me summarize what you told me about your symptoms; so you cough every night at least five times, you have constant pain in the left leg (developed by authors, adapted from the studies by Ong et al, 1995; Cavaco and Roter, 2010; Roter et al, 1997; and Cegala, 1997 [1,35-37])

The next step is dedicated to scripting the 2 CA interventions designed for patients with chronic obstructive pulmonary disease (COPD). We focused on COPD as a chronic condition, as the number of people affected by COPD continues to rise inexorably on a global scale, hence causing hardships among the affected and a tremendous financial burden on health care systems [44]. We based the content of the CA dialog on the teaching workbooks of “Living well with COPD,” an evidence-based disease-specific self-management program originally developed at the Montreal Chest Institute in collaboration with the Respiratory Health Network of the Fonds de la Recherche en Santé du Quebec and Boehringer Ingelheim [45]. We decided to model the first day of this hypothetical patient-CA intervention as it incorporates relevant interaction categories (eg, introduction and patient education) of a comprehensive disease management intervention. In a two-step process, we first scripted a base-case CA interaction whose interaction style was as neutral as possible. In the second step, we followed our systematically developed specifications of communication components and corresponding frequency-level characteristics for the 2 interaction styles to develop 2 distinct CA scripts. This means that we reverse-engineered the labeling process of utterances depicted in the RIAS methodology to induce either a paternalistic or a deliberative interaction style into the base-case intervention, that is, editing, adding, or deleting separate sentences, parts of sentences, or terms to differentiate the frequency of communication items of the base-case CA interaction. We ensured that both the scripts were approximately of the same length, with 38 conversational turns (with 96 individual messages) in the deliberative and 32 (with 85 individual messages) in the paternalistic version. The 2 scripts differed in 40 instances, whereas this number included differences in the level of a single word as the smallest adjustable part of a sentence. These discrepancies are caused by the characteristics of the 2 interaction styles, and these discrepancies are to ensure the realism of the conversation flow. For instance, we defined that a deliberative interaction style has a high frequency of emotional talk around a partnership. In one of the first conversational turns, we thus scripted “Okay. Understood, then we are ready to start.” as a potential possibility of an answer in the deliberative script versus solely “Okay. Understood.” in the paternalistic script. The conversation tree had only one level of branch points and was then reverted to the main conversational flow. The average overall reading duration was 13.5 min for the deliberative and 12 min for the paternalistic version. The scripts were written in German. The 2 intervention variations were then presented to senior medical experts working in the pulmonary division of a European University Hospital, who assessed and confirmed realism. Excerpts of the scripts are shown and discussed in the Results section, and the complete scripts can be found in Multimedia Appendix 3. The intervention was purely text based without any visual or spoken cues to reduce any bias toward visual CA design features, such as gender, age, or visual appearance [33,46]. Hence, we named the CA Robo, a gender-neutral name.

### Validation of CAs’ Interaction Styles

In the second phase, we validated the interaction styles and assessed whether participants engaging with 1 of the 2 CAs could identify the correct interaction style.

#### Experimental Design and Procedure

We conducted a closed, between-subjects web-based experiment, in which the participants were randomly prompted to engage with a CA that follows either a deliberative or paternalistic interaction style. Following the Checklist for Reporting of Results of Internet E-Surveys [47], we report on the design, procedure, and results of this experiment. Qualtrics software (Qualtrics XM), a software- and web-based survey and data collection platform, was used to design the experiment and to randomly assign participants to 1 of the 2 CAs. Collect.chat, a commercially available chatbot software, was used to develop chatbot dialogs. The CA was integrated into the Qualtrics HTML using an iframe. Before starting the web-based experiment, we tested its usability and technical functionality. The experiment was conducted between March 27 and April 11, 2020. The questionnaire comprised a total of 35 questions distributed over 8 pages (between 1 and 17 items per page). The respondents were not able to review and change their answers.

The experimental procedure was set up as follows: first, we informed the participants about the structure and length of the survey, its potential risks and confidentiality, data protection, and possible uses of the data. We also provided contact details of the investigators in the case of questions and comments. After receiving informed consent, we checked the participation conditions, such as being aged above 18 years and speakers of German language. We then queried a set of sociodemographic questions (age, gender, mother tongue, and education). Second, the participants were presented with a short and easy-to-comprehend scenario description that depicted the day of a patient with COPD who started using a CA (for details, refer to Multimedia Appendix 4), an established approach in health care for the investigation of specific aspects in a medical context [48,49]. The scenario prompted participants to put themselves in the position of a patient with COPD to be able to relate to the subsequent interaction with the CA [50,51]—a targeted health outcome was not included. We ensured that the necessary heterogeneity of participants was much better than that with a limited set of patients who are often homogeneous on the key characteristics such as age due to their shared medical condition (eg, COPD becomes clinically noticeable only from the age of 40-50 years [52]) by applying a scenario description with healthy participants instead of conducting the experiment with actual patients. Third, after presenting the scenario, the interaction with the CA started, which we embedded in a separate, dedicated page of the survey. The interaction was purely text based and comprised a prescripted dialog based on the 2 developed scripts, that is, one for the paternalistic and another for the deliberative interaction style. Participants chose between 1 to 3 predefined answer options. They interacted with the respective CA on their individual pace, with no supervision or guidance from the researchers. Fourth, after the interaction, the participants answered questions about the interaction style. Here, the participants were asked to choose which of the following 2 statements better described their perception of Robo’s interaction style with them: (1) Robo decides paternally, based on objective principles, or (2) Robo and I discuss and decide together. The former refers to a paternalistic, whereas the latter to a deliberative interaction style.

#### Participants

A priori power analysis was conducted using the R package (version 3.5.2) power analysis [53]. To identify a medium effect (*r*=0.30) in a chi-square test of independence at an α level of .05 and statistical power of 0.80, a total of 88 participants were required. Inclusion criteria of this study are as follows: participants had to be of aged above 18 years and German-speaking. The participants were recruited via email and social media through an anonymous link. The participants who were invited to participate in this study were from the authors’ academic institutions, networks, and cooperating partners, and participation was entirely voluntary, with no incentives offered. We intended to actively drive heterogeneity in the sample to ensure external validity. We recruited a total of 112 participants. Of these 112 participants, 24 did not complete the questionnaire and were thus excluded. Thus, the final sample comprised 88 persons (42/88, 48% female, mean age 31.5 years, SD 10.1 years), resulting in a completion rate of 79%. Table 2 shows an overview of the demographics of the participants. On average, the participants needed 29 min for the whole experiment, including the questionnaire and the CA interaction.

**Table 2:** Demographic characteristics of participants (n=88).

Characteristic		Value
Age in years, mean (SD)		31.5 (10.1)
Gender (female), n (%)		42 (48)
**Education, n (%)**		
	<High school	15 (17)
	High school	9 (10)
	University degree	64 (73)

#### Data Analysis

To test whether the participants correctly identified the interaction type of the CAs, we applied a chi-square test of independence for a 2×2 contingency table and calculated the related phi coefficient using the statistical software R (version 3.5.2). The phi coefficient is a measure of the strength of the association between 2 binary variables. If it is positive, that is, the 2 variables are positively associated, then most of the data fall along the diagonal cells. To acknowledge the validation of the CAs as successful, we set 2 criteria based on the previous literature: (1) a significant chi-square test statistic with at least 80% of participants correctly identifying their assigned respective CA in each condition [43] and (2) a positive phi coefficient of at least a medium effect size (*r*_φ_≥0.30) [54].

## Results

**Figure 1:**
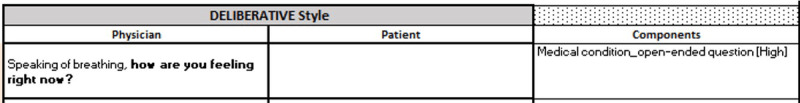
An example of the developed script for the deliberative version of the conversational agent (CA) Robo is shown here. The column labeled “Components” depicts the applied communication item and its respective communication frequency for the respective interaction style. The column labeled “Physician” describes what the CA Robo says, whereas the column “Patient” shows the reply options of the participant.

**Figure 2:**
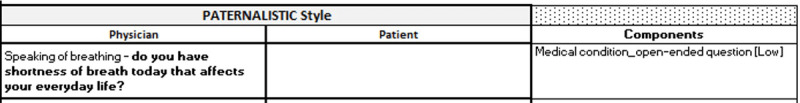
An example of the developed script for the paternalistic version of the conversational agent (CA) Robo is shown here. The column labeled “Components” depicts the applied communication item and its respective communication frequency for the respective interaction style. The column labeled “Physician” describes what the CA Robo says, whereas the column “Patient” shows the reply options of the participant.

The results of the development of the deliberative and paternalistic interaction styles are presented in this section, whereas the development process of the interaction styles themselves is described in the Methods section. The described process resulted in 2 written scripts that were used for scripting the paternalistic and deliberative versions of the CA Robo. The scripts further included the answer options of the participants, the underlying communication items, and the applied communication frequency for each utterance. Figures 1 and 2 show an example of how the different interaction styles were induced into the CA script. The scripts were initially developed in German. This conversation has been translated into English by the authors for this study.

**Figure 3:**
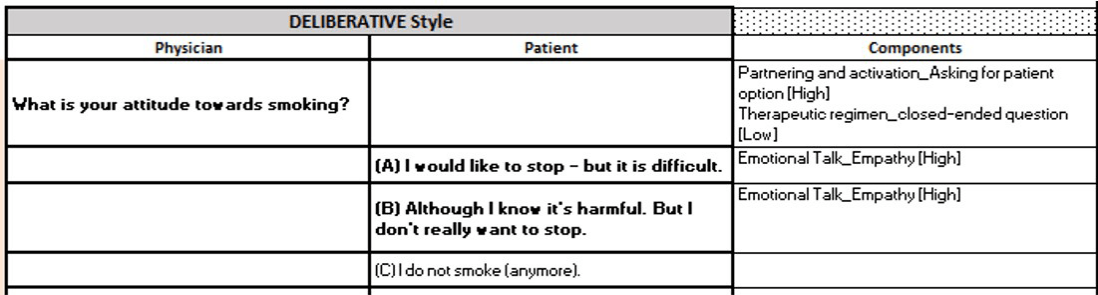
An example of the developed script for the deliberative version of the conversational agent Robo is shown here. Here, the answer options for patients, as depicted in the column “Patient,” deviated between the 2 conditions.

**Figure 4:**
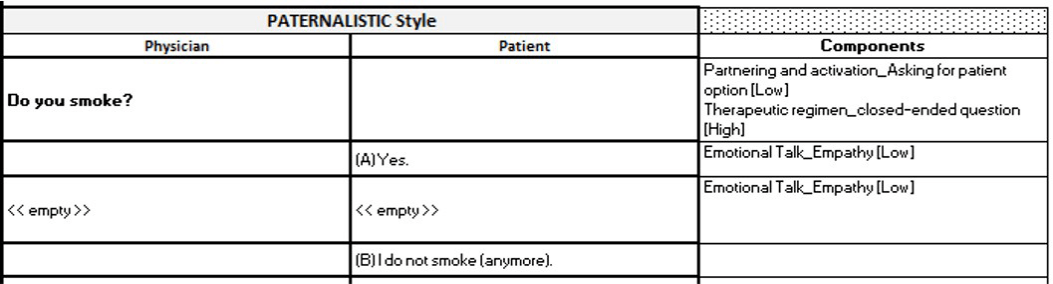
An example of the developed script for the paternalistic version of the conversational agent Robo is shown here. Here, the answer options for the patients, as depicted in the column “Patient,” deviated between the 2 conditions.

Figures 3 and 4 show an example of the interaction style scripts where the answers of the patients needed to deviate between the 2 interaction styles. This is because the interaction would have felt artificial when the same answer options would have been used for both the interaction styles.

**Figure 5:**
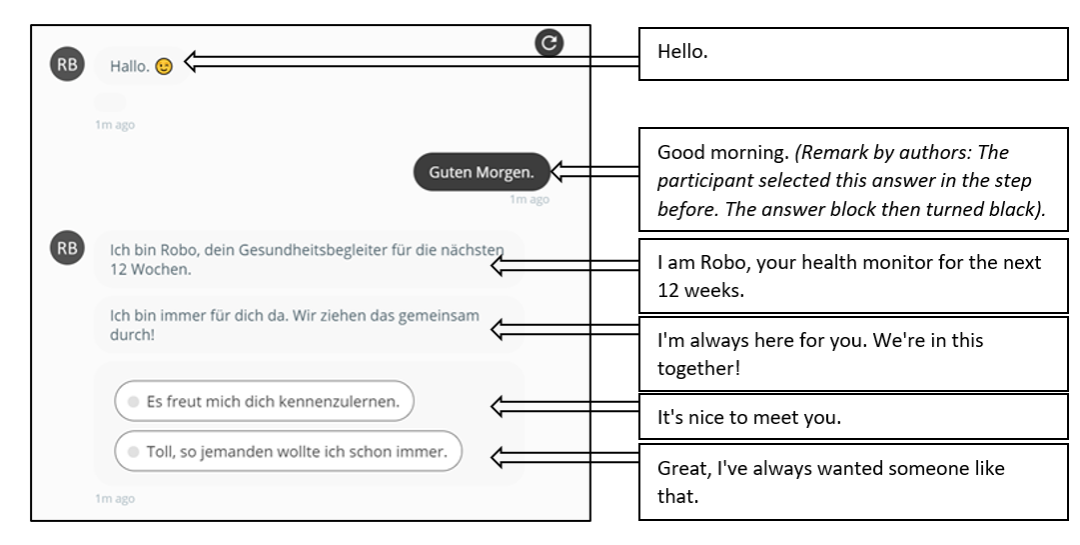
An exemplary conversation snapshot between the conversational agent Robo and a participant. This figure depicts the start of the interaction, here applying the deliberative interaction style. The interaction was conducted in German; the authors added the English translations in the callouts for this study.

The developed scripts were implemented into the technical CA environment. In the web-based experiment, the participants could only see the utterances of the CA Robo (noted in the column “Physician” of Figures 1-4) and their possible answer options (noted in the column “Patient” of Figures 1-4). Figure 5 shows an exemplary conversational turn between a participant and the CA Robo as implemented in the web-based experiment.

We describe the results of the web-based experiment in the following section. On the basis of a 2×2 contingency table, a chi-square test of independence was performed to examine the relationship between the randomly assigned CA type and the correct identification of CA type by the participants (Table 3). The relationship between these variables was significant (*X*^2^_1,88_=38.2; *P*<.001), thereby indicating unequal frequency distributions between the cells. The associated phi coefficient of correlation was r_φ_=0.68, corresponding to a large effect size [54]. Participants in both the paternalistic and deliberative conditions correctly identified their respective CA type more than 80% of the time: the probability of recognizing the paternalistic interaction style when it was, in fact, paternalistic was 37/(37+7)=84%. The same was true for the deliberative condition. Thus, based on the previously defined set of criteria (significant results of the chi-square test, 80% of participants correctly identified the respective CA in each condition, and phi coefficient *r*_φ_≥0.30), the validation of the interaction styles was successful.

**Table 3:** Contingency table of assigned and identified conversational agent interaction styles^a^.

Identified by participants	Random assignment	
	P^b^	D^c^
P^b^	37	7
D^c^	7	37
	37/(37+7)=0.84	37/(37+7)=0.84

^a^2x2 contingency table. The performance of 2 developed conversational agents was assessed by comparing the categories randomly assigned to participants by the experimenters with participants’ own perception which category the assigned conversational agent belonged to. The type of conversational agent assigned by the experimenters is designated as paternalistic (P) or deliberative (D) and is listed above the 2x2 table.

^b^P: paternalistic.

^c^D: deliberative.

## Discussion

### Principal Findings

We developed an approach to induce deliberative and paternalistic interaction styles into a purely text-based patient-CA conversation [5]. It was developed for chronic health care applications against the background of the high relevance of chronic diseases and the patients’ support potential of CAs. To our knowledge, this is the first attempt to develop and evaluate 2 different interaction styles for the interaction between a CA and a human user in a chronic health care context. This study successfully proves that humans can correctly identify and label an induced interaction style under experimental conditions. When randomly assigned, participants in both the paternalistic and deliberative experimental conditions correctly identified the interaction style in more than 80% of the cases. The procedure is based on modifying the frequency of communication items that are adopted from human-human medical interactions. Although we focused on the deliberative and paternalistic interaction styles, we expect the underlying methodology to be also applicable for inducing other interaction styles.

In the context of chronic diseases, a rising number of medical interventions is already based on the application of CAs [24,28,30,55-57]. However, studies investigating the interaction styles between humans and CAs are still scarce. Examples include a range of applications from health care to real estate and implicitly assume a default interaction between the agent and user [58-60]. In our opinion, this assumption of a standardized human-agent interaction disregards patients’ preferences of different interaction styles as derived from human-human interactions. We addressed this shortcoming with our study; our approach allows the repeated and independent development of any patient-agent medical conversation with a deliberative and paternalistic interaction style. We therefore enable other researchers to develop and test these interaction styles in different health care contexts. This is the first but important building block to develop personalized CAs by better understanding and investigating which interaction styles offered by CAs are relevant in which health care situations (eg, acute vs chronic care situations or disease-specific situations), for which patient group (eg, for patient segmentation), and at which stage in the course of the disease and diseases-related dynamics (high- vs low-pain situations). By considering these additional factors in future work, we assume that the effectiveness of CAs can be further increased by deploying more personalized CA-based conversations that fit the specific medical context and the patient situation at hand.

As indicated above, the quality and effectiveness of the interaction between patients and physicians has already been established as decisive for treatment outcomes in human-human medical encounters [61,62]. This patient-physician interaction is acknowledged as a dynamic process in which personal preferences for the most effective communication can change over time [61,63]. Skilled physicians can adapt their interaction style to each patient and his or her situation, thereby improving the treatment outcome [63]. Until now, such dynamic and individual adaption of patient-physician interactions can only be realized within a human-human context. However, it would be beneficial to have digitized solutions that are capable of the same dynamic alterations in the form of adaptive CAs for providing on-demand individualized medical support. Such solutions would yield hope for increased treatment adherence and subsequently improved medical outcomes, especially within the context of chronic diseases that often require prolonged medical oversight and support. The first part of developing such dynamic interactive CAs is to build their ability to provide more than one interaction style for communication with human users. In this paper, we provided this first stage by showing that 2 different interaction styles can be successfully developed, implemented into a CA-patient communication, and correctly identified and labeled by humans in the context of chronic disease health care.

### Strengths and Limitations

This study has several strengths. By adapting the RIAS [35], a validated and widely used coding scheme for medical dialogs, we ensured an objective approach to identify the key communication items that induce the interaction styles. We detailed and refined the procedure by integrating the knowledge of novice and experienced medical experts, thereby ensuring a broad range of expertise in terms of patient-physician interaction behavior taught in medical school as well as practical experience with patients. We further measured the IRR to objectively calculate the agreement between the experts and our assessment and to select the most important items. Moreover, we tested the developed scripts with additional medical experts before scripting the CAs used in the web-based experiment.

A limitation of this study is that participants were all German-speaking and based in Switzerland, Germany, and Austria. Other languages might have different requirements regarding essential communication items for representing a deliberative and paternalistic interaction style. Cultural differences with respect to power distance, individualism, and uncertainty avoidance, as analyzed by Hofstede [64], might also play a significant role. Another limitation is the focus on one specific chronic condition. Although we consider our methodology sufficient to model medical conversations for other disease conditions, we only tested it in the context of COPD. Patients with other conditions—acute or chronic—might have other demands for their interaction with a digital CA. In addition, the CA in our experiment was rule based with prescripted answer possibilities. Although this was necessary to control the experimental conditions between the 2 interaction conditions, there is a rising number of artificial intelligence (AI)–based CAs for health care apps. These agents do not rely on prescripted conversations but often allow for natural interaction using unconstrained written, spoken, or visual input [24,25,56]. The applicability of our procedure for AI-based agents should be evaluated in future research.

### Suggestions for Future Research

In general, we advise future research in the field of digital health care to put a stronger focus on the consideration of different interaction styles between human users and CAs. We propose that such CAs are based on our approach for inducing either a paternalistic or deliberative interaction style. It still must be determined which of the 2 interaction styles is the best situational fit for an individual engaging with a CA. In the following step, dynamic CAs can adapt their interaction style to both the personal and situational circumstances of individual users, much like human physicians are already able to do [61,63]. We suggest evaluating the implementation of human-agent interaction styles in different medical contexts, such as various acute and chronic conditions, as well as with different health care goals such as diagnosis, treatment, or patient education.

Furthermore, CAs with varying interaction styles should be developed in various languages and in different cultures. This would allow the investigation of the role of language and culture in interaction preferences. In addition, there might be more than the described 2 interaction styles that are of interest in the medical context. For example, Emanuel and Emanuel [5] also described an informative and interpretative interaction style. These could also be adapted for and integrated into CAs to develop a broader range of options for patient-CA interactions, for example, in the context of the information about invasive examination or interventions.

Moreover, we only modeled and tested the first day of an interaction between a patient and a CA. It would be interesting to see the effect of longer interventions. This is especially relevant for chronic conditions, as they require ongoing treatment and accompanying digital interventions for potentially the entire treatment period.

Furthermore, we suggest the implementation of our approach to the architecture of an AI-based CA to evaluate its applicability for this type of technology. Finally, because our CA was purely text based, it would be interesting to see what role other communication forms such as visualizations, embodiment, or spoken interaction play for the induction of particular interaction styles.

## Appendix

Multimedia Appendix 1 Overview items.

Multimedia Appendix 2 Inter-rater reliability item selection.

Multimedia Appendix 3 Scripts of conversational agents in German and English.

Multimedia Appendix 4 Scenario description in German and English.

## Abbreviations

AI: artificial intelligence
CA: conversational agent
COPD: chronic obstructive pulmonary disease
IRR: inter-rater reliability
RIAS: Roter Interaction Analysis System

## References

[1] Ong L, de Haes J, Hoos A, Lammes F (1995). Doctor-patient communication: a review of the literature. Soc Sci Med.

[2] Kaplan SH, Greenfield S, Ware JE (1989). Assessing the effects of physician-patient interactions on the outcomes of chronic disease. Med Care.

[3] Flückiger C, Del Re AC, Wampold BE, Horvath AO (2018). The alliance in adult psychotherapy: a meta-analytic synthesis. Psychotherapy (Chic).

[4] Di Blasi Z, Harkness E, Ernst E, Georgiou A, Kleijnen J (2001). Influence of context effects on health outcomes: a systematic review. Lancet.

[5] Emanuel EJ, Emanuel LL (1992). Four models of the physician-patient relationship. J Am Med Assoc.

[6] Heritage J, Maynard DW (2006). Problems and prospects in the study of physician-patient interaction: 30 years of research. Annu Rev Sociol.

[7] Borza LR, Gavrilovici C, Stockman R (2015). Ethical models of physician--patient relationship revisited with regard to patient autonomy, values and patient education. Rev Med Chir Soc Med Nat Iasi.

[8] Montori VM, Gafni A, Charles C (2006). A shared treatment decision-making approach between patients with chronic conditions and their clinicians: the case of diabetes. Health Expect.

[9] Blanchard CG, Labrecque MS, Ruckdeschel JC, Blanchard EB (1988). Information and decision-making preferences of hospitalized adult cancer patients. Soc Sci Med.

[10] Benbassat J, Pilpel D, Tidhar M (1998). Patients' preferences for participation in clinical decision making: a review of published surveys. Behav Med.

[11] Say R, Murtagh M, Thomson R (2006). Patients' preference for involvement in medical decision making: a narrative review. Patient Educ Couns.

[12] Gibson PG, Talbot PI, Toneguzzi RC (1995). Self-management, autonomy, and quality of life in asthma. Population Medicine Group 91C. Chest.

[13] Strull WM (1984). Do patients want to participate in medical decision making?. J Am Med Assoc.

[14] Hvidberg MF, Johnsen SP, Glümer C, Petersen KD, Olesen AV, Ehlers L (2016). Catalog of 199 register-based definitions of chronic conditions. Scand J Public Health.

[15] VAN DEN BOS GA (1995). The burden of chronic diseases in terms of disability, use of health care and healthy life expectancies. Eur J Public Health.

[16] Han MK, Quibrera PM, Carretta EE, Barr RG, Bleecker ER, Bowler RP, Cooper CB, Comellas A, Couper DJ, Curtis JL, Criner G (2017). Frequency of exacerbations in patients with chronic obstructive pulmonary disease: an analysis of the SPIROMICS cohort. Lancet Respir Med.

[17] Kvedar JC, Fogel AL, Elenko E, Zohar D (2016). Digital medicine's march on chronic disease. Nat Biotechnol.

[18] Wagner EH (1998). Chronic disease management: what will it take to improve care for chronic illness?. Eff Clin Pract.

[19] Frey U, Suki B (2008). Complexity of chronic asthma and chronic obstructive pulmonary disease: implications for risk assessment, and disease progression and control. Lancet.

[20] Chobanian AV, Bakris GL, Black HR, Cushman WC, Green LA, Izzo JL, Jones DW, Materson BJ, Oparil S, Wright JT, Roccella EJ, Joint National Committee on Prevention‚ Detection‚ Evaluation‚Treatment of High Blood Pressure. National Heart‚ Lung‚Blood Institute, National High Blood Pressure Education Program Coordinating Committee (2003). Seventh report of the Joint National Committee on Prevention, Detection, Evaluation, and Treatment of High Blood Pressure. Hypertension.

[21] Stone NJ (2008). Nonpharmacologic management of mixed dyslipidemia associated with diabetes mellitus and the metabolic syndrome: a review of the evidence. Am J Cardiol.

[22] van der Wal MH, van Veldhuisen DJ, Veeger NJ, Rutten FH, Jaarsma T (2010). Compliance with non-pharmacological recommendations and outcome in heart failure patients. Eur Heart J.

[23] Wise RA, Tashkin DP (2007). Optimizing treatment of chronic obstructive pulmonary disease: an assessment of current therapies. Am J Med.

[24] Laranjo L, Dunn AG, Tong HL, Kocaballi AB, Chen J, Bashir R, Surian D, Gallego B, Magrabi F, Lau AY, Coiera E (2018). Conversational agents in healthcare: a systematic review. J Am Med Inform Assoc.

[25] Schachner T, Keller R, Wangenheim FV (2020). Artificial intelligence-based conversational agents for chronic conditions: systematic literature review. J Med Internet Res.

[26] Bickmore TW, Mitchell SE, Jack BW, Paasche-Orlow MK, Pfeifer LM, Odonnell Julie (2010). Response to a Relational Agent by Hospital Patients with Depressive Symptoms. Interact Comput.

[27] Bickmore TW, Picard RW (2005). Establishing and maintaining long-term human-computer relationships. ACM Trans Comput-Hum Interact.

[28] Hauser-Ulrich S, Künzli H, Meier-Peterhans D, Kowatsch T (2020). A smartphone-based health care chatbot to promote self-management of chronic pain (SELMA): pilot randomized controlled trial. JMIR Mhealth Uhealth.

[29] Fadhil A, Gabrielli S, Kessler F (2017). Addressing challenges in promoting healthy lifestyles: the Al-chatbot approach. Proceedings of the 11th EAI International Conference on Pervasive Computing Technologies for Healthcare.

[30] Ma T, Sharifi H, Chattopadhyay D (2019). Virtual Humans in Health-Related Interventions: A Meta-Analysis. Extended Abstracts of the 2019 CHI Conference on Human Factors in Computing Systems.

[31] Crutzen R, Peters GY, Portugal SD, Fisser EM, Grolleman JJ (2011). An artificially intelligent chat agent that answers adolescents' questions related to sex, drugs, and alcohol: an exploratory study. J Adolesc Health.

[32] Kang SH, Feng AW, Leuski A, Casas D, Shapiro A (2015). The effect of an animated virtual character on mobile chat interactions. Proceedings of the 3rd International Conference on Human-Agent Interaction.

[33] ter Stal S, Kramer LL, Tabak M, op den Akker H, Hermens H (2020). Design features of embodied conversational agents in eHealth: a literature review. Int J Hum Comput Stud.

[34] Reach G (2013). Patient autonomy in chronic care: solving a paradox. Patient Prefer Adherence.

[35] Cavaco A, Roter D (2010). Pharmaceutical consultations in community pharmacies: utility of the Roter Interaction Analysis System to study pharmacist-patient communication. Int J Pharm Pract.

[36] Roter DL, Stewart M, Putnam SM, Lipkin M, Stiles W, Inui TS (1997). Communication patterns of primary care physicians. J Am Med Assoc.

[37] Cegala DJ (1997). A study of doctors' and patients' communication during a primary care consultation: implications for communication training. J Health Commun.

[38] Ong LM, Visser MR, Kruyver IP, Bensing JM, van den Brink-Muinen A, Stouthard JM, Lammes FB, de Haes JC (1998). The Roter Interaction Analysis System (RIAS) in oncological consultations: psychometric properties. Psychooncology.

[39] Detmar SB, Muller MJ, Wever LD, Schornagel JH, Aaronson NK (2001). The patient-physician relationship. Patient-physician communication during outpatient palliative treatment visits: an observational study. J Am Med Assoc.

[40] Charles C, Gafni A, Whelan T (1997). Shared decision-making in the medical encounter: what does it mean? (or it takes at least two to tango). Soc Sci Med.

[41] Street RL (1992). Communicative styles and adaptations in physician-parent consultations. Soc Sci Med.

[42] Williams S, Weinman J, Dale J (1998). Doctor-patient communication and patient satisfaction: a review. Fam Pract.

[43] McHugh ML (2012). Interrater reliability: the kappa statistic. Biochem Med (Zagreb).

[44] Mannino DM, Buist AS (2007). Global burden of COPD: risk factors, prevalence, and future trends. Lancet.

[45] Bourbeau J, Julien M, Maltais F, Rouleau M, Beaupré A, Bégin R, Renzi P, Nault D, Borycki E, Schwartzman K, Singh R, Collet J, Chronic Obstructive Pulmonary Disease axis of the Respiratory Network Fonds de la Recherche en Santé du Québec (2003). Reduction of hospital utilization in patients with chronic obstructive pulmonary disease: a disease-specific self-management intervention. Arch Intern Med.

[46] McDonnell M, Baxter D (2019). Chatbots and gender stereotyping. Interact Comput.

[47] Eysenbach G (2004). Improving the quality of Web surveys: the Checklist for Reporting Results of Internet E-Surveys (CHERRIES). J Med Internet Res.

[48] Offredy M, Kendall S, Goodman C (2008). The use of cognitive continuum theory and patient scenarios to explore nurse prescribers' pharmacological knowledge and decision-making. Int J Nurs Stud.

[49] Offredy M (2002). Decision-making in primary care: outcomes from a study using patient scenarios. J Adv Nurs.

[50] Bahari SF (2012). Qualitative versus quantitative research strategies: contrasting epistemological and ontological assumptions. Jurnal Teknologi.

[51] Bell E, Bryman A, Harley B (2018). Business research methods. Research Methods.

[52] Postma DS, Bush A, van den Berge M (2015). Risk factors and early origins of chronic obstructive pulmonary disease. Lancet.

[53] Fan FY (2017). Power Analysis in Experimental Design. cran.R-Project.

[54] Cohen J (1988). Statistical Power Analysis for the Behavioral Sciences.

[55] Kocaballi AB, Berkovsky S, Quiroz JC, Laranjo L, Tong HL, Rezazadegan D, Briatore A, Coiera E (2019). The personalization of conversational agents in health care: systematic review. J Med Internet Res.

[56] Montenegro JLZ, da Costa CA, da Rosa Righi R (2019). Survey of conversational agents in health. Expert Syst Appl.

[57] Kramer J, Künzler F, Mishra V, Smith S, Kotz D, Scholz U, Fleisch E, Kowatsch T (2020). Which components of a smartphone walking app help users to reach personalized step goals? Results from an optimization trial. Ann Behav Med.

[58] Bickmore T, Gruber A, Picard R (2005). Establishing the computer-patient working alliance in automated health behavior change interventions. Patient Educ Couns.

[59] Cassell J, Bickmore T (2003). Negotiated collusion: modeling social language and its relationship effects in intelligent agents. User Model User-adapt Interact.

[60] Kidd C (2008). Designing for Long-Term Human-Robot Interaction and Application to Weight Loss. Program in Media Arts and Sciences, School of Architecture and Planning at the MASSACHUSETTS INSTITUTE OF TECHNOLOGY.

[61] Angus D, Watson B, Smith A, Gallois C, Wiles J (2012). Visualising conversation structure across time: insights into effective doctor-patient consultations. PLoS One.

[62] Street RL, Makoul G, Arora NK, Epstein RM (2009). How does communication heal? Pathways linking clinician-patient communication to health outcomes. Patient Educ Couns.

[63] Loughlan C, Mutrie N (2014). Conducting an exercise consultation: guidelines for health professionals. J Inst Heal Educ.

[64] Hofstede G (2016). National cultures in four dimensions: a research-based theory of cultural differences among nations. Int Stud Manag Organ.

