# Supplementary material for: Deliberative and Paternalistic Interaction Styles for Conversational Agents in Digital Health: Procedure and Validation Through a Web-Based Experiment
Source: J Med Internet Res. 2021 Jan 29;23(1):e22919. doi: 10.2196/22919 (PMC7880814; doi:10.2196/22919)
Supplement: Multimedia Appendix 1 [file jmir_v23i1e22919_app1.pdf]

Functional Grouping			Category				Cluster		
**Functional Group (RIAS_NEW 2010)**	*Functional Group (RIAS_OLD 1997)*	*Functional group_Ong 1995 (allocati*	*Category_Ong 1995 (allocation TS)*	*Category_RIAS OLD (1997)*	*Category (category_communication behavior category)_Ong 1998*	**Category_Communication behavior_RIAS NEW 2010**	**Cluster_Communication behavior_RIAS NEW 2010**	**Example Statement**	**References**
Data-gathering skills	Content	Instrumental behavior	Exchange of information_seeking	Question asking	Question-asking_instrumental	Open-ended question	Medical condition	"What can you tell me about the pain?" (Cavaco & Roter, 2010, p. 143)	Cavaco & Roter 2010. Roter 1997. Ong 1995: Ong 1998: Cegala 1997
Data-gathering skills	Process	Instrumental behavior	Exchange of information_seeking	Question asking	Question-asking_instrumental	Open-ended question	Therapeutic regimen	How are your symptoms developing since you take the new pills?	Cavaco & Roter 2010. Roter 1997. Ong 1995: Ong 1998: Cegala 1997
Data-gathering skills		Instrumental behavior	Exchange of information_seeking		Question-asking_instrumental	Open-ended question	Lifestyle and self-care	"What are you doing to keep yourself healthy"? (Cavaco & Roter, 2010, p. 143)	Cavaco & Roter 2010, Ong 1995; Ong 1998; Cegala 1997
Data-gathering skills	Content	Instrumental behavior	Exchange of information_seeking	Question asking	Question-asking_instrumental	Open-ended question	Psychosocial topics / feelings	How has the situation at home with your sick parent developing?	Cavaco & Roter 2010, Roter 1997, Ong 1995; Ong 1998; Cegala 1997
Data-gathering skills	Content	Instrumental behavior	Exchange of information_seeking	Question asking	Question-asking_instrumental	Closed-ended questions	Medical condition	Do you cough at night?	Cavaco & Roter 2010, Roter 1997, Ong 1995; Ong 1998; Cegala 1997
Data-gathering skills	Content	Instrumental behavior	Exchange of information_seeking	Question asking	Question-asking_instrumental	Closed-ended questions	Therapeutic regimen	Did you reduce your cigarette consumation to max. 10 cigarettes / day as di	Cavaco & Roter 2010, Roter 1997, Ong 1995; Ong 1998; Cegala 1997
Data-gathering skills		Instrumental behavior	Exchange of information_seeking		Question-asking_instrumental	Closed-ended questions	Lifestyle and self-care	Do you go to the gym once a week?	Cavaco & Roter 2010, Ong 1995; Ong 1998; Cegala 1997
Data-gathering skills	Content	Instrumental behavior	Exchange of information_seeking	Question asking	Question-asking_instrumental	Closed-ended questions	Psychosocial topics / feelings	Is your parent still sick?	Cavaco & Roter 2010, Roter 1997, Ong 1995; Ong 1998; Cegala 1997
Patient Education and Counselling Skills	Content	Instrumental behavior	Making treatment-related decision	Biomedical information	Counselling (persuasion)_instrumental	Biomedical information	About medical condition	Your lung function is lower than the last time.	Cavaco & Roter 2010, Roter 1997, Ong 1995; Ong 1998
Patient Education and Counselling Skills	Content	Instrumental behavior	Making treatment-related decision	Biomedical information	Counselling (persuasion)_instrumental	Biomedical information	Therapeutic regimen	You need to do this exercise once a day for 15 minutes.	Cavaco & Roter 2010, Roter 1997, Ong 1995; Ong 1998
Patient Education and Counselling Skills		Instrumental behavior	Making treatment-related decision		Counselling (persuasion)_instrumental	Lifestyle and self-care information		Spending time with other COPD patients is a good thing as you can exchange	Cavaco & Roter 2010, Ong 1995; Ong 1998
Patient Education and Counselling Skills	Content	Instrumental behavior	Exchange of information_seeking	Psychosocial exchange	Counselling (persuasion)_instrumental	Psychosocial Exchange about problems of daily living, issues about social relations, feelings, emotions		It is important to talk about your worries regarding your condition.	Cavaco & Roter 2010, Roter 1997, Ong 1995; Ong 1998
Relationship Skills	Affect	Affective behavior	Creation of good inter-personal relationship	Positive talk	Verbal attentiveness_affective	Positive Talk	Agreements / Showing understanding	Yes. that's the correct way to do the exercise. I understand it is hard to do th	Cavaco & Roter 2010, Roter 1997, Ong 1995; Ong 1998
Relationship Skills	Affect	Affective behavior	Creation of good inter-personal relationship	Positive talk	Social behavior_affective	Positive Talk	Friendly Jokes and Laughter	See you later alligator!	Cavaco & Roter 2010, Roter 1997, Ong 1995; Ong 1998
Relationship Skills	Affect	Affective behavior	Creation of good inter-personal relationship	Positive talk	Social behavior_affective	Positive Talk	Approvals/Compliments	You're doing great with this exercise, good job!	Cavaco & Roter 2010, Roter 1997, Ong 1995; Ong 1998
Relationship Skills	Affect	Affective behavior	*Creation of good inter-personal relationship*	*Negative talk*	Negative talk_affective	Negative Talk	Disagreements	No, I would not recommend you going to this specific patient group meeting	Cavaco & Roter 2010, Roter 1997, Ong 1995; Ong 1998
Relationship Skills	Affect	Affective behavior	*Creation of good inter-personal relationship*	Negative talk	Negative talk_affective	Negative Talk	Disapproval and *criticisms* and *corrections*	That's not how much you should take from this pill; you have to be careful to	Cavaco & Roter 2010, Roter 1997, Ong 1995; Ong 1998
Relationship Skills	Affect	Affective behavior	Creation of good inter-personal relationship	Social talk	Social behavior_affective	Social talk (non-medical, chit-chat, *personal remarks*)		So, what are your plans for summer vacation - anthing plannend already?	Cavaco & Roter 2010, Roter 1997, Ong 1995; Ong 1998
Relationship Skills		Affective behavior	Creation of good inter-personal relationship		Showing concern_affective	Emotional talk	Concerns / Worry	I'm concerned about your lung function.	Cavaco & Roter 2010, Ong 1995; Ong 1998; Detmar et al. 2001
Relationship Skills		Affective behavior	Creation of good inter-personal relationship		Showing concern_affective	Emotional talk	Reassurance / Optimism	Your arm will feel better soon, no worries!	Cavaco & Roter 2010, Roter 1997, Ong 1995; Ong 1998
Relationship Skills		Affective behavior	Creation of good inter-personal relationship		Social behavior_affective	Emotional talk	Legitimate	"Anyone would worry if they felt as bad as you do." (Cavaco & Roter, 2010, p.	Cavaco & Roter 2010, Roter 1997, Ong 1995; Ong 1998
Relationship Skills		Affective behavior	Creation of good inter-personal relationship		Verbal attentiveness_affective	Emotional talk	Empathy	I can see how worried you are from hearing these results of your lung test.	Cavaco & Roter 2010, Roter 1997, Ong 1995; Ong 1998; Detmar et al. 2001
Relationship Skills		Affective behavior	Creation of good inter-personal relationship			Emotional talk	Partnership	"We'll get through this together." (Cavaco & Roter, 2010, p. 143)	Cavaco & Roter 2010, Ong 1995
Partnering skills	Process	Affective behavior	Exchange of information_verifying	Facilitation		Partnering and activation	Asking for patient opinion	Do you want to bring your husband to the next session?	Cavaco & Roter 2010, Roter 1997, Ong 1995; Cegala 1997
Partnering skills	Process	Affective behavior	Exchange of information_verifying	Facilitation		Partnering and activation	Asking for understanding	Can you follow all my instructions?	Cavaco & Roter 2010, Roter 1997, Ong 1995; Cegala 1997; Detmar et al. 2001
Partnering skills	Process	Affective behavior	Exchange of information_verifying	Facilitation	Verbal attentiveness_affective	Partnering and activation	Paraphrase and interpretation	Ok. let me summarize what you told me about your symptoms: so you cough	Cavaco & Roter 2010, Roter 1997, Ong 1995; Ong 1998; Cegala 1997; Detmar et al. 2001
Partnering skills		Affective behavior	Exchange of information_verifying			Partnering and activation	Cues of interest (back-channel)	Ok, mhm, continue,...	Cavaco & Roter 2010, Ong 1995; Ong 1998; Cegala 1997
Partnering skills	Process	Instrumental behavior	Exchange of information_giving	Orientation	Directions_instrumental	Orientation (directions, instructions)		I'll test your lung function now. Please take off your shirt and sit over there a	Cavaco & Roter 2010, Roter 1997, Ong 1995; Ong 1998; Cegala 1997; Detmar et al. 2001

Hierarchy: Functional Group (FG) > Category > Cluster
