# Supplementary material for: Deliberative and Paternalistic Interaction Styles for Conversational Agents in Digital Health: Procedure and Validation Through a Web-Based Experiment
Source: J Med Internet Res. 2021 Jan 29;23(1):e22919. doi: 10.2196/22919 (PMC7880814; doi:10.2196/22919)
Supplement: Multimedia Appendix 2 [file jmir_v23i1e22919_app2.pdf]

__Inter-item IRR and Item selection__

Complete item list

Code: 1 = chosen, 0 = not chosen

#	Communication component [cluster_category]	Rater1	Rater2	Rater3	Rater4	Rater5	Rater6	% Agreement per item / coder
1	Medical condition_open-ended question	1	1	1	1	1		83%
2	Therapeutic regimen_open-ended question	0	1	1	1	1	1	83%
3	Lifestyle and self-care_open-ended question	1	1			1	1	67%
4	Psychosocial topics / feelings_open-ended question	0	0			1		17%
5	Medical condition_closed-ended question	0	0	1			1	33%
6	Therapeutic regimen_closed-ended question	1	1	1	1	1	1	100%
7	Lifestyle and self-care_closed-ended question	0	0			1	1	33%
8	Psychosocial topics / feelings_closed-ended question	0	0	1	1			33%
9	Lifestyle and self-care information	0	0		1	1	1	50%
10	Psychosocial Exchange about problems of daily living, issues about social relations, feelings, emotions	1	1	1	1	1	1	100%
11	Positive Talk_Friendly Jokes and Laughter	1	1			1		50%
12	Positive Talk_Approvals/Compliments	1	0		1		1	50%
13	Social talk (non-medical, chit-chat, personal remarks)	1	1	1	1			67%
14	Emotional Talk_Concerns / Worry	1	1			1	1	67%
15	Emotional Talk_Reassurance / Optimism	1	1	1	1	1	1	100%
16	Emotional Talk_Legitimate	1	1	1	1			67%
17	Emotional Talk_Empathy	1	1	1	1	1	1	100%
18	Emotional Talk_Partnership	1	1	1	1			67%
19	Partnering and activation_Asking for patient opinion	1	1	1	1	1	1	100%
20	Partnering and activation_Asking for understanding	1	1	1	1	1	1	100%
21	Partnering and activation_Paraphrase and interpretation	1	1	1	1	1	1	100%
22	Partnering and activation_Cues of interest (back-channel)	0	0	1			1	33%
	sum	15	15	15	15	15	15	
	check	ok	ok	ok	ok	ok	ok	

Approach item selection based on IRR

I. Item selection based on 80% cut-off rate
