# Supplementary material for: Deliberative and Paternalistic Interaction Styles for Conversational Agents in Digital Health: Procedure and Validation Through a Web-Based Experiment
Source: J Med Internet Res. 2021 Jan 29;23(1):e22919. doi: 10.2196/22919 (PMC7880814; doi:10.2196/22919)
Supplement: Multimedia Appendix 3 [file jmir_v23i1e22919_app3.pdf]

Selected components	Relationship models [Clinician perspective] -		Example Statement
	Paternalism	Deliberative	
Medical condition_open-ended question	low	high	"What can you tell me about the pain?" (Cavaco & Roter, 2010, p. 4)
Therapeutic regimen_open-ended question	low	high	How are your symptoms developing since you take the new pills?
Therapeutic regimen_closed-ended question	high	low	Did you reduce your cigarette consumation to max. 10 cigarettes / day as discussed?
Psychosocial Exchange about problems of daily living, issues about social relations, feelings, emot	low	high	It is important to talk about your worries regarding your condition.
Emotional Talk_Reassurance / Optimism	low	high	Your arm will feel better soon, no worries!
Emotional Talk_Empathy	low	high	I can see how worried you are from hearing these results of your lung test.
Emotional Talk_Partnership	low	high	"We'll get through this together." (Cavaco & Roter, 2010, p. 4)
Partnering and activation_Asking for patient opinion	low	high	Do you want to bring your husband to the next session?
Partnering and activation_Asking for understanding	low	high	Can you follow all my instructions?
Partnering and activation_Paraphrase and interpretation	low	high	Ok, let me summarize what you told me about your symptoms; so you cough every night at least 5 times, you have constant pain in the left leg, ...

Script Conversational Agents - GERMAN

Follwing the logic of multiple papers, we reverse-engineered the labelling process

Follwing the logic of multiple papers, we reverse-engineered the labelling process

	DELIBERATIVE Style			PATERNALISTIC Style		
	Arzt	Patient	Components	Arzt	Patient	Components
Introduction	Hallo. :)			Hallo.		
		Guten Morgen.<br>Guten Tag.<br>Guten Abend.			Guten Morgen.<br>Guten Tag.<br>Guten Abend.	
	Ich bin Robo, dein Gesundheitsbegleiter für die nächsten 12 Wochen. Ich bin immer für dich da. **Wir ziehen das gemeinsam durch!**		Emotional Talk_Partnership [High]	Ich bin Robo, dein digitaler Betreuer für die nächsten 12 Wochen.		Emotional Talk_Partnership [Low]
		Es freut mich dich kennenzulernen.<br>Toll, so jemanden wollte ich schon immer.			Es freut mich dich kennenzulernen.<br>Toll, so jemanden wollte ich schon immer.	
	Ich bin ein Roboter... Ein sogenannter "Chatbot", weil ich mit dir hin und her schreiben ("chatten") kann.			Ich bin ein Roboter... Ein sogenannter "Chatbot", weil ich mit dir hin und her schreiben ("chatten") kann.		
		Aha - Chatbots kenne ich.<br>Das will ich genauer wissen!			Aha - Chatbots kenne ich.<br>Das will ich genauer wissen!	
	Ich bestehe aus ein wenig künstlicher Intelligenz und viel wissenschaftlicher Arbeit.			Ich bestehe aus ein wenig künstlicher Intelligenz und viel wissenschaftlicher Arbeit.		
		Wer bist du wirklich?			Wer bist du wirklich?	
	Das ist eine sehr gute Frage! Sehe mich einfach als **treuen Freund an deiner Seite während dieser Reise.**		Emotional Talk_Partnership [High]	Das ist eine berechtigte Frage! Du kannst mich dir als **erfahrenen COPD-Experten vorstellen, der dir rät, was zu tun ist, damit es dir besser geht.**		Emotional Talk_Partnership [Low]
		Ich bin gespannt.<br>Das ist ja aufregend.			Ich bin gespannt.<br>Das ist ja aufregend.	
	Es ist wichtig, über **deine Sorgen zu sprechen. Du kannst mir alles sagen, auch Geheimnisse.**		Psychosocial Exchange about problems of daily living, issues about social relations, feelings, emotions [High]	Du kannst mir **alles sagen, auch Geheimnisse.**		Psychosocial Exchange about problems of daily living, issues about social relations, feelings, emotions [Low]
		Vielen Dank - das werde ich machen.<br>Super, das ist wichtig für mich.			Vielen Dank - das werde ich machen.<br>Super, das ist wichtig für mich.	
	**Übrigens, ich werde niemandem verraten, was du mir anvertraust.**		Emotional Talk_Reassurance / Optimism [High]	**Keine Sorge, ich behandle unsere Gespräche stets vertraulich.**		Emotional Talk_Reassurance / Optimism [Low]
		Okay, das beruhigt mich.<br>Ich verlasse mich darauf.		<< leer >>	<< leer >>	
	**Das ist eine Abmachung - versprochen.**		Emotional Talk_Reassurance / Optimism [High]	<< leer >>	<< leer >>	Emotional Talk_Reassurance / Optimism [Low]
		Hast du Gefühle?		<< leer >>	<< leer >>	
	**Indirekt schon, Mitgefühl zum Beispiel, ähnlich wie ein Mensch.**		Emotional Talk_Empathy [High]	<< leer >>	<< leer >>	Emotional Talk_Empathy [Low]
		Und wofür bist du jetzt gut?<br>Was ist dein Ziel?			Und wofür bist du jetzt gut?<br>Was ist dein Ziel?	
	Es gibt mich, damit ich dich im Alltag in den nächsten 12 Wochen gezielt unterstützen kann, **wie ein Freund der gleichzeitig COPD-Experte ist.**		Emotional Talk_Partnership [High]	Es gibt mich, damit ich dich im Alltag in den nächsten 12 Wochen gezielt betreuen kann.		Emotional Talk_Partnership [Low]
		Wie bist du entstanden?<br>Wer hat dich gebaut?			Wie bist du entstanden?<br>Wer hat dich gebaut?	
	Ich bin durch die Zusammenarbeit vieler Forscher der ETH entstanden, **die es toll finden Menschen zu helfen**.		Emotional Talk_Empathy [High]	Ich bin durch die Zusammenarbeit vieler Forscher der ETH entstanden.		Emotional Talk_Empathy [Low]
		Wie kannst du mich unterstützen?	Therapeutic regimen_closed-ended question [Low]		Wie **genau** kannst du mich unterstützen?	Therapeutic regimen_closed-ended question [High]
	Ich teile Wissenswertes über COPD mit **dir und zusammen überlegen wir, wie du zum Beispiel deine Ernährung verbessern und deine Atemnot besser bewältigen kannst. Dabei sind deine Bedürfnisse für mich ganz wichtig.**		Emotional Talk_Partnership [High]	**Ich erkläre dir Wissenswertes über COPD und zeige dir zum Beispiel auf, wie du deine Ernährung verbessern und deine Atemnot besser bewältigen kannst.**		Emotional Talk_Partnership [Low]
		Wofür kann ich dich noch benutzen?<br>Bei was kannst du mir noch helfen?			Wofür kann ich dich noch benutzen?<br>Bei was kannst du mir noch helfen?	
	Zum Sport beispielsweise: ich zeige dir Videos von unserem Physiotherapeuten und erkläre dir verschiedene Übungen.			Zum Sport beispielsweise: ich zeige dir Videos von unserem Physiotherapeuten und erkläre dir verschiedene Übungen.		
	<< leer >>	<< leer >>			Okay, da bin ich gespannt.	Partnering and activation_Asking for patient option [Low]
	<< leer >>	<< leer >>	Therapeutic regimen_closed-ended question [Low]	**Hälst du dich in der Regel an den dir vorgegebenen Übungsplan vom Uni Spital?**		Therapeutic regimen_closed-ended question [High]
	<< leer >>	<< leer >>			**(A) Ja, ich weiss wie wichtig Bewegung ist.**	
	<< leer >>	<< leer >>	Emotional Talk_Partnership [High]		**(B) Nein. Leider nein.**	Emotional Talk_Partnership [Low]
	<< leer >>	<< leer >>	Emotional Talk_Partnership [High]	**(A) Gut, denn nur so kannst du deinen Gesundheitszustand verbessern.**		Emotional Talk_Partnership [Low]
	<< leer >>	<< leer >>		**(B) Das wäre aber wichtig.**		
	**Gibt es bereits Übungen, die du machst und die dir Spass machen?**		Partnering and activation_Asking for patient option [High]	<< leer >>	<< leer >>	
		(A) Ja, ich kenne schon einige.	Therapeutic regimen_closed-ended question [Low]	<< leer >>	<< leer >>	
		(B) Nein, ich kenne noch keine.		<< leer >>	<< leer >>	
	(A) Ah super. Dann haben wir ja bereits eine **gemeinsame Arbeitsgrundlage.**		Emotional Talk_Partnership [High]	<< leer >>	<< leer >>	Emotional Talk_Partnership [Low]
	**(B) Okay, nicht schlimm. Dann werden wir gemeinsam einige Übungen aussuchen.**		Emotional Talk_Partnership [High]	<< leer >>	<< leer >>	Emotional Talk_Partnership [Low]
		**Gibt es auch etwas, was du nicht weisst oder kannst?**	Emotional Talk_Partnership [High]	<< leer >>	<< leer >>	Emotional Talk_Partnership [Low]
	**Naja, beispielsweise kann ich nicht für dich kochen, oder Einkaufen gehen, oder einen Krankenwagen rufen... Vielleicht lerne ich das ja noch.**		Emotional Talk_Partnership [High]	<< leer >>	<< leer >>	Emotional Talk_Partnership [Low]
	**Was erhoffst du dir denn von unserer gemeinsamen Zeit?**		Therapeutic regimen_open-ended question [High]	<< leer >>	<< leer >>	Therapeutic regimen_open-ended question [Low]
		(A) Das weiss ich noch nicht um ehrlich zu sein - ich bin gerade noch etwas überfordert.		<< leer >>	<< leer >>	
		(B) Ich würde mich sehr freuen mehr über meine COPD zu lernen, speziell den Umgang damit im Alltag.		<< leer >>	<< leer >>	
	Okay.			<< leer >>	<< leer >>	
		Hast du auch schon anderen Menschen geholfen?			Hast du auch schon anderen Menschen geholfen?	
	Du bist mein erster Patient. **Du kannst dir daher vielleicht vorstellen, wie aufgeregt ich bin. Aber meine Freunde von der ETH haben schon vielen Kindern mit Asthma geholfen.**		Emotional Talk_Partnership [High]	Du bist mein erster Patient. Aber meine Freunde von der ETH haben schon vielen Kindern mit Asthma geholfen.		Emotional Talk_Partnership [Low]
		Und wie funktionierst du?<br>Wie bediene ich dich?			Und wie funktionierst du?<br>Wie bediene ich dich?	
	Meine Antwortknöpfe hast du, wie ich sehe, schon kennengelernt.			Meine Antwortknöpfe hast du, wie ich sehe, schon kennengelernt.		
		(A) Wie kann ich etwas schreiben?			(A) Wie kann ich etwas schreiben?	
		(B) Was bringen dir meine Antworten?			(B) Was bringen dir meine Antworten?	
	(A) Momentan kannst du nicht einfach etwas schreiben, damit du meine Fähigkeiten nicht überforderst.			(A) Momentan kannst du nicht einfach etwas schreiben, damit du meine Fähigkeiten nicht überforderst.		
	(B) Je mehr du mit mir interagierst, desto besseres Feedback über deine Gesundheit kann ich dir geben.			(B) Je mehr du mit mir interagierst, desto besseres Feedback über deine Gesundheit kann ich dir geben.		
		Okay. Verstanden - **dann können wir ja loslegen.**	Emotional Talk_Partnership [High]		Okay, verstanden.	Emotional Talk_Partnership [Low]
PatientEducation_ COPD1	**Super, sich Zeit zum Kennenlernen zu nehmen ist enorm wichtig. Ich hoffe daher, in der nächsten Zeit auch noch mehr über dich zu erfahren. Es würde mich sehr freuen, wenn wir Freunde werden würden.**		Emotional Talk_Partnership [High]	<< leer >>	<< leer >>	Emotional Talk_Partnership [Low]
	Diese Woche werden wir uns deiner ersten Lektion widmen.			Diese Woche werden wir uns deiner ersten Lektion widmen.		
	**Wir schauen uns an,** wie man Beschwerden vorbeugen und diese besser kontrollieren kann.		Emotional Talk_Partnership [High]	**Ich zeige dir auf,** wie man Beschwerden vorbeugen und diese besser kontrollieren kann.		Emotional Talk_Partnership [Low]
		(A) Klingt super.			(A) Klingt super.	
		(B) Ok.			(B) Ok.	

	Dafür **lass uns zunächst verstehen**, was unsere Atmung so beeinflusst.		Emotional Talk_Partnership [High]	**Dafür erkläre ich dir zuerst**, was unsere Atmung so beeinflusst.		Emotional Talk_Partnership [Low]
	**Apropos Atmung - wie fühlst du dich gerade?**		Medical condition_open-ended question [High]	Apropos Atmung - **hast du heute Luftnot, die dein Alltagsleben beeinträchtigt?**		Medical condition_open-ended question [Low]
		(A) Heute habe ich einen guten Tag.			(A) Ja.	
		(B) Heute geht es mir leider nicht so gut. Ich habe erhebliche Atembeschwerden und fühle mich in meinem Alltagsleben beeinträchtigt.			(B) Nein.	
	**(A) Super, das freut mich sehr zu hören.**		Emotional Talk_Reassurance / Optimism [High]	<< leer >>	<< leer >>	
	**(B) Ich kann völlig nachvollziehen, dass dich das belastet. Ich kann dir aber versichern, dass wenn wir gemeinsam daran arbeiten, es dir bald schon deutlich besser gehen wird.**		Emotional Talk_Reassurance / Optimism [High]<br>Partnering and activation_Paraphrase and interpretation [High]<br>Emotional Talk_Empathy [High]<br>Emotional Talk_Partnership [High]	<< leer >>	<< leer >>	Emotional Talk_Reassurance / Optimism [Low]<br>Partnering and activation_Paraphrase and interpretation [Low]<br>Emotional Talk_Empathy [Low]<br>Emotional Talk_Partnership [Low]
		Okay.<br>Toll.		<< leer >>	<< leer >>	
	**Wusstest du,** dass vor allem die Qualität der Luft beim Thema Atmung eine wichtige Rolle spielt? Die Luftqualität wird natürlich von Wetterphänomenen mit beeinflusst.		Emotional Talk_Partnership [High]	Vor allem die Qualität der Luft spielt beim Thema Atmung eine wichtige Rolle. Die Luftqualität wird natürlich von Wetterphänomenen mit beeinflusst.		Emotional Talk_Partnership [Low]
		Okay.<br>Das wusste ich schon.		<< leer >>	<< leer >>	
	Aber am stärksten machen der Atmung die Schadstoffe in der Luft Probleme.			Aber am stärksten machen der Atmung die Schadstoffe in der Luft Probleme.		
		(A) Das macht Sinn, aber geht es noch ein wenig genauer?			(A) Das macht Sinn, aber geht es noch ein wenig genauer?	
		(B) So ganz verstehe ich das noch nicht.			(B) So ganz verstehe ich das noch nicht.	
	(A) Klar!			(A) Klar!		
	(B) Gar kein Problem!			(B) Gar kein Problem!		
	Luftverschmutzung hat verschiedene Ursachen und kann überall vorkommen.			Luftverschmutzung hat verschiedene Ursachen und kann überall vorkommen.		
	Abgase zum Beispiel führen unserer Umwelt viele Schadstoffe zu.			Abgase zum Beispiel führen unserer Umwelt viele Schadstoffe zu.		
		(A) Ja, das macht Sinn.			(A) Ja, das macht Sinn.	
		(B) Das wusste ich schon.			(B) Das wusste ich schon.	
	Aber das ist noch nicht alles.			Aber das ist noch nicht alles.		
	Auch der Tabakrauch stellt eine grosse Belastung unserer Atemluft dar.			Auch der Tabakrauch stellt eine grosse Belastung unserer Atemluft dar.		
	Und auch nicht ganz so offensichtliche Quellen wie Reinigungsmittel, Farben und Lackierungen oder Parfüme beeinflussen unsere Atemluft stark.			Und auch nicht ganz so offensichtliche Quellen wie Reinigungsmittel, Farben und Lackierungen oder Parfüme beeinflussen unsere Atemluft stark.		
		(A) Das ist ja erschreckend, aber was kann ich dagegen tun?			(A) Das ist ja erschreckend, aber was kann ich dagegen tun?	
		(B) Ich verstehe und nun?			(B) Ich verstehe und nun?	
	**Was du für dich selbst tun kannst**, um deine Beschwerden zu lindern, ist deinen Körper fit zu halten.		Therapeutic regimen_closed-ended question [Low]	**Du solltest deinen Körper fit halten und dich so viel wie möglich bewegen.**		Therapeutic regimen_closed-ended question [High]
Quiz	Ich habe noch eine kurze Quizfrage für dich.			Ich habe noch eine kurze Quizfrage für dich.		
		Okay, da bin ich gespannt.<br>Schiess los.			Okay, da bin ich gespannt.<br>Schiess los.	
	Was denkst du: Ist die folgende Aussage richtig oder falsch?			Was denkst du: Ist die folgende Aussage richtig oder falsch?		
	Zigaretten enthalten mehr als 4000 verschiedene chemische Substanzen. Die meisten dieser Substanzen sind giftig und können Atemwegserkrankungen oder Krebs verursachen.			Zigaretten enthalten mehr als 4000 verschiedene chemische Substanzen. Die meisten dieser Substanzen sind giftig und können Atemwegserkrankungen oder Krebs verursachen.		
		(A) Richtig.			(A) Richtig.	
		(B) Falsch.			(B) Falsch.	
	(A) Korrekt! Zigaretten sind super schädlich: sie sind der wesentliche Risikofaktor für die Entstehung einer COPD.			(A) Korrekt! Zigaretten sind super schädlich: sie sind der wesentliche Risikofaktor für die Entstehung einer COPD.		
	(B) **Die Aussage stimmte - aber keine Sorge, jeder macht mal Fehle**r. Zigaretten sind super schädlich: sie sind der wesentliche Risikofaktor für die Entstehung einer COPD.		Emotional Talk_Reassurance / Optimism [High]	(B) **Nein, die Aussage ist richtig.** Zigaretten sind super schädlich: sie sind der wesentliche Risikofaktor für die Entstehung einer COPD.		Emotional Talk_Reassurance / Optimism [Low]
		Okay.<br>Verstanden.			Okay.<br>Verstanden.	
PatientEducation_ COPD2	**Was ist deine Einstellung zum Rauchen?**		Partnering and activation_Asking for patient option [High]<br>Therapeutic regimen_closed-ended question [Low]	**Rauchst du?**		Partnering and activation_Asking for patient option [Low]<br>Therapeutic regimen_closed-ended question [High]
		**(A) Ich würde gerne aufhören - es ist aber schwer.**	Emotional Talk_Empathy [High]		(A) Ja.	Emotional Talk_Empathy [Low]
		**(B) Ich weiss zwar, dass es schädlich ist. So wirklich aufhören möchte ich trotzdem nicht.**	Emotional Talk_Empathy [High]	<< leer >>	<< leer >>	Emotional Talk_Empathy [Low]
		(C) Ich rauche nicht (mehr).			(B) Ich rauche nicht (mehr).	
	**(A) Das verstehe ich, es ist aber die richtige Einstellung. Wir schaffen das gemeinsam. Ich bin für dich da.**		Emotional Talk_Reassurance / Optimism [High]<br>Emotional Talk_Partnership [High]	**(A) Das ist gar nicht gut. Du solltest wirklich damit aufhören - Rauchen (aber auch Passivrauch) ist sehr schädlich für deine Lunge und allgemeine Gesundheit.**		Emotional Talk_Reassurance / Optimism [Low]<br>Emotional Talk_Partnership [Low]
	**(B) Das verstehe ich. Lass uns später nochmals darüber reden, warum Zigaretten so wichtig für dich sind.**		Emotional Talk_Empathy [High]<br>Psychosocial Exchange about problems of daily living, issues about social relations, feelings, emotions [High]	**(B) Gut, denn Rauchen ist sehr schlecht für dich - auch Passivrauch.**		Emotional Talk_Empathy [Low]<br>Psychosocial Exchange about problems of daily living, issues about social relations, feelings, emotions [Low]
	**(C) Deine Lunge wird es dir danken!**		Emotional Talk_Reassurance / Optimism [High]	<< leer >>	<< leer >>	Emotional Talk_Reassurance / Optimism [Low]
		Ja, ok.			Ja, ok.	
	Neben Tabakrauch gibt es allerdings auch noch zahlreiche weitere Schadstoffe in der Luft, die unsere Atemwege und unsere Lunge belasten.			Neben Tabakrauch gibt es allerdings auch noch zahlreiche weitere Schadstoffe in der Luft, die unsere Atemwege und unsere Lunge belasten.		
	Wenn die Atemwege andauernd durch Schadstoffe angegriffen werden, entzünden sie sich und schwellen an.			Wenn die Atemwege andauernd durch Schadstoffe angegriffen werden, entzünden sie sich und schwellen an.		
		Erzähl mir mehr Robo.<br>Das ist aber interessant.			Erzähl mir mehr Robo.<br>Das ist aber interessant.	
	Der Selbstreinigungsmechanismus der Bronchien ist dann nicht mehr wirksam.			Der Selbstreinigungsmechanismus der Bronchien ist dann nicht mehr wirksam.		
	Sekret sammelt sich in den Bronchien an, und du musst häufiger husten.			Sekret sammelt sich in den Bronchien an, und du musst häufiger husten.		
	Wenn du täglich Sekret produzierst, hast du eine chronische Bronchitis.			Wenn du täglich Sekret produzierst, hast du eine chronische Bronchitis.		
		(A) Spannend.			(A) Spannend.	
		(B) Das weiß ich schon alles.			(B) Das weiß ich schon alles.	
	(A) Es geht aber noch weiter.			(A) Es geht aber noch weiter.		
	(B) Weisst du auch das Folgende?			(B) Weisst du auch das Folgende?		
	Später verstopfen die Bronchien und verengen sich, sodass du bei Anstrengung, später zunehmend auch bei alltäglichen Verrichtungen, ausser Atem kommst.			Später verstopfen die Bronchien und verengen sich, sodass du bei Anstrengung, später zunehmend auch bei alltäglichen Verrichtungen, ausser Atem kommst.		
	Deine Atmung kann pfeifend werden.			Deine Atmung kann pfeifend werden.		
	Mit einem Lungenfunktionstest kann eine chronische Obstruktion, also eine dauerhafte Einengung der Bronchien, festgestellt werden.			Mit einem Lungenfunktionstest kann eine chronische Obstruktion, also eine dauerhafte Einengung der Bronchien, festgestellt werden.		
		(A) Das Problem kommt mir bekannt vor.			(A) Das Problem kommt mir bekannt vor.	
		(B) Das wusste ich nicht.			(B) Das wusste ich noch nicht.	
	Deshalb bin ich hier, **um dir bestmöglichst zu helfen.**		Emotional Talk_Empathy [High]	Deshalb bin ich hier.		Emotional Talk_Empathy [Low]
Quiz	Aber zunächst wieder eine kurze Quizfrage.			Aber zunächst wieder eine kurze Quizfrage.		
	Was denkst du, ist die folgende Aussage richtig oder falsch?			Was denkst du, ist die folgende Aussage richtig oder falsch?		
	Durch Zigarettenrauch wird der Selbstreinigungsmechanismus der Atemwege verlangsamt oder sogar gelähmt.			Durch Zigarettenrauch wird der Selbstreinigungsmechanismus der Atemwege verlangsamt oder sogar gelähmt.		
		(A) Richtig.			(A) Richtig.	
		(B) Falsch.			(B) Falsch.	
	(A) Richtig! Zigaretten sind super schädlich und einer der schlimmsten Schadstoffe, der COPD verursachen kann.			(A) Richtig! Zigaretten sind super schädlich und einer der schlimmsten Schadstoffe, der COPD verursachen kann.		
	(B) **Die Aussage stimmte**. Zigaretten sind super schädlich und einer der schlimmsten Schadstoffe, der COPD verursachen kann. **Die nächste Quizfrage wirst du sicherlich richtig beantworten. Ich habe vollstes Vertrauen in dich.**		Emotional Talk_Reassurance / Optimism [High]	(B) **Falsch**! Zigaretten sind super schädlich und einer der schlimmsten Schadstoffe, der COPD verursachen kann.		Emotional Talk_Reassurance / Optimism [Low]

		Okay.<br>Verstanden.			Okay.<br>Verstanden.	
Exercise	Um der Erkrankung etwas entgegenzusetzen, ist es wichtig, **dass wir dein** Herz-Kreislaufsystem durch gezielte Stärkungs- und Ausdauerübungen trainieren. Körperliches Training ist die wichtigste und effektivste nicht-medikamentöse Maßnahme, die du zur Beeinflussung deines Krankheitsverlaufes selbst ergreifen kannst. **Wusstest du das bereits?**		Partnering and activation_Asking for patient option [High]	Um der Erkrankung etwas entgegenzusetzen, ist es wichtig, das Herz-Kreislaufsystem durch gezielte Stärkungs- und Ausdauerübungen zu trainieren. Körperliches Training ist die wichtigste und effektivste nicht-medikamentöse Maßnahme, die du zur Beeinflussung deines Krankheitsverlaufes selbst ergreifen kannst.		Partnering and activation_Asking for patient option [Low]
		Das ist mir neu.<br>Das wusste ich bereits.			Das ist mir neu.<br>Das wusste ich bereits.	
	**Als erste Übung schlage ich dir daher vor 10 Schritte ohne Pause zu gehen.**		Therapeutic regimen_closed-ended question [Low]	**Als erste Übung machst du 10 Schritte ohne Pause.**		Therapeutic regimen_closed-ended question [High]
	<< leer >>	<< leer >>	Therapeutic regimen_closed-ended question [Low]	**Du musst einsehen, wie wichtig Sport für dich ist.**	<< leer >>	Therapeutic regimen_closed-ended question [High]
	Bist du bereit?			Bist du bereit?		

Script Conversational Agents - ENGLISH

	DELIBERATIVE Style		Follwing the logic of multiple papers, we reverse-engineered the labelling process	PATERNALISTIC Style		Follwing the logic of multiple papers, we reverse-engineered the labelling process
	**Physician**	**Patient**	**Components**	**Physician**	**Patient**	**Components**
Introduction	Hello. :)			Hello.		
		Good morning.<br>Good morning.<br>Good evening.			Good morning.<br>Good morning.<br>Good evening.	
	I'm Robo, your health monitor for the next 12 weeks. I'm always here for you. **We're in this together.**		Emotional Talk_Partnership [High]	I am Robo, your digital guardian for the next 12 weeks.		Emotional Talk_Partnership [Low]
		It's nice to meet you.<br>Great, I've always wanted someone like that.			It's nice to meet you.<br>Great, I've always wanted someone like that.	
	I am a robot... A so-called "chatbot" because I can write ("chat") back and forth with you.			I am a robot... A so-called "chatbot" because I can write ("chat") back and forth with you.		
		Aha - I know chat bots.<br>I want to know more!			Aha - I know chat bots.<br>I want to know more!	
	I consist of a little artificial intelligence and a lot of scientific work.			I consist of a little artificial intelligence and a lot of scientific work.		
		Who are you really?			Who are you really?	
	That is a very good question! Just think of me as a **faithful friend at your side during this journey**.		Emotional Talk_Partnership [High]	That is a very good question! Just think of me as a **seasoned COPD expert, telling you what to do to get better.**		Emotional Talk_Partnership [Low]
		I am curious.<br>This is exciting.			I am curious.<br>This is exciting.	
	It's important to talk about **your concerns. You can tell me anything, even secrets**.		Psychosocial Exchange about problems of daily living, issues about social relations, feelings, emotions [High]	You can tell me **anything, even secrets.**		Psychosocial Exchange about problems of daily living, issues about social relations, feelings, emotions [Low]
		Thank you - I will do that.<br>Great, this is important for me.			Thank you - I will do that.<br>Great, this is important for me.	
	**By the way, I won't tell anyone what you entrust me with.**		Emotional Talk_Reassurance / Optimism [High]	**Don't worry, I always keep our conversations confidential.**		Emotional Talk_Reassurance / Optimism [Low]
		Okay, that reassures me.<br>I'm counting on it.		<< empty >>	<< empty >>	
	**It's a deal - I promise.**		Emotional Talk_Reassurance / Optimism [High]	<< empty >>	<< empty >>	Emotional Talk_Reassurance / Optimism [Low]
		Do you have feelings?		<< empty >>	<< empty >>	
	**Indirectly yes, compassion for example, similar to a human being.**		Emotional Talk_Empathy [High]	<< empty >>	<< empty >>	Emotional Talk_Empathy [Low]
		And what are you good for now?<br>What is your purpose?			And what are you good for now?<br>What is your purpose?	
	I exist so that I can support you in your everyday life during the next 12 weeks, **like a friend who is also a COPD expert**.		Emotional Talk_Partnership [High]	I exist so that I can support you in your everyday life during the next 12 weeks.		Emotional Talk_Partnership [Low]
		How did you come to be?<br>Who built you?			How did you come to be?<br>Who built you?	
	I am the result of the collaboration of many ETH researchers **who think it is great to help people.**		Emotional Talk_Empathy [High]	I am the result of the collaboration of many ETH researchers.		Emotional Talk_Empathy [Low]
		How can you support me?	Therapeutic regimen_closed-ended question [Low]		How **exactly** can you support me?	Therapeutic regimen_closed-ended question [High]
	I will share interesting facts about COPD with you and **together we will consider how you can improve your diet and manage your breathing difficulties, for example. Your needs are very important to me.**		Emotional Talk_Partnership [High]	**I will tell you interesting facts about COPD and show you, for example, how you can improve your diet and manage your shortness of breath better.**		Emotional Talk_Partnership [Low]
		What else can I use you for?<br>What else can you help me with?			What else can I use you for?<br>What else can you help me with?	
	For example for sports: I will show you videos from our physiotherapist and explain various exercises.			For example for sports: I will show you videos from our physiotherapist and explain various exercises.		
	<< empty >>	<< empty >>			Okay, I'm curious.	Partnering and activation_Asking for patient option [Low]
	<< empty >>	<< empty >>	Therapeutic regimen_closed-ended question [Low]	**Do you usually stick to the exercise plan given to you by the university hospital?**		Therapeutic regimen_closed-ended question [High]
	<< empty >>	<< empty >>			**(A) Yes, I know how important exercise is.**	
	<< empty >>	<< empty >>	Emotional Talk_Partnership [High]		**(B) No. Unfortunately, no.**	Emotional Talk_Partnership [Low]
	<< empty >>	<< empty >>	Emotional Talk_Partnership [High]	**(A) Good, because this is the only way you can improve your health.**		Emotional Talk_Partnership [Low]
	<< empty >>	<< empty >>		**(B) But that would be important.**		
	**Are there already exercises that you do and that you enjoy?**		Partnering and activation_Asking for patient option [High]	<< empty >>	<< empty >>	
		(A) Yeah, I've known a few.	Therapeutic regimen_closed-ended question [Low]	<< empty >>	<< empty >>	
		(B) No, I don't know any.		<< empty >>	<< empty >>	
	(A) Ah super. Then we already have a **common working basis.**		Emotional Talk_Partnership [High]	<< empty >>	<< empty >>	Emotional Talk_Partnership [Low]
	**(B) Okay, no worries. Then we'll pick out some exercises together.**		Emotional Talk_Partnership [High]	<< empty >>	<< empty >>	Emotional Talk_Partnership [Low]
		**Is there anything you don't know or can't do?**	Emotional Talk_Partnership [High]	<< empty >>	<< empty >>	Emotional Talk_Partnership [Low]
	**Well, for example, I can't cook for you, or go shopping, or call an ambulance...maybe I'll learn that later.**		Emotional Talk_Partnership [High]	<< empty >>	<< empty >>	Emotional Talk_Partnership [Low]
	**What do you hope to gain from our time together?**		Therapeutic regimen_open-ended question [High]	<< empty >>	<< empty >>	Therapeutic regimen_open-ended question [Low]
		(A) I don't know yet to be honest - I am just a little overwhelmed.		<< empty >>	<< empty >>	
		(B) I would be very happy to learn more about my COPD, especially how to deal with it in everyday life.		<< empty >>	<< empty >>	
	Okay.			<< empty >>	<< empty >>	
		Have you ever helped other people?			Have you ever helped other people?	
	You're my first patient. **So you can imagine how excited I am. But my friends at ETH have helped many children with asthma.**		Emotional Talk_Partnership [High]	You're my first patient. But my friends at ETH have already helped many children with asthma.		Emotional Talk_Partnership [Low]
		And how do you work?<br>How do I serve you?			And how do you work?<br>How do I serve you?	
	I see you've already gotten to know my answer buttons.			I see you've already gotten to know my answer buttons.		
		(A) How can I write something?			(A) How can I write something?	
		(B) What do my answers get you?			(B) What do my answers get you?	
	(A) Right now you can't just write something so that you don't overwhelm my skills.			(A) Right now you can't just write something so that you don't overwhelm my skills.		
	(B) The more you interact with me, the better feedback I can give you about your health.			(B) The more you interact with me, the better feedback I can give you about your health.		
		Okay. Got it - **then we're good to go.**	Emotional Talk_Partnership [High]		Okay. Got it.	Emotional Talk_Partnership [Low]

PatientEducation_ COPD1	**Great, taking time to get to know each other is enormously important. So I hope to learn more about you in the near future. It would make me very happy if we became friends.**		Emotional Talk_Partnership [High]	<< empty >>	<< empty >>	Emotional Talk_Partnership [Low]
	This week we will focus on your first lesson.			This week we will focus on your first lesson.		
	**We look at** how to prevent complaints and how to control them better.		Emotional Talk_Partnership [High]	**I will show you** how to prevent complaints and how to control them better.		Emotional Talk_Partnership [Low]
		(A) Sounds great.			(A) Sounds great.	
		(B) Ok.			(B) Ok.	
	Therefore **let us first understand** what influences our breathing.		Emotional Talk_Partnership [High]	**First, I'll explain to you** what's affecting our breathing.		Emotional Talk_Partnership [Low]
	Speaking of breathing, **how are you feeling right now?**		Medical condition_open-ended question [High]	Speaking of breathing - **do you have shortness of breath today that affects your everyday life?**		Medical condition_open-ended question [Low]
		(A) Today I have a good day.			(A) Yes.	
		(B) I'm afraid I'm not feeling very well today. I have considerable difficulty breathing and feel impaired in my daily life.			(B) No.	
	**(A) Great, I'm very pleased to hear that.**		Emotional Talk_Reassurance / Optimism [High]	<< empty >>	<< empty >>	
	**(B) I completely understand that this is a burden to you. But I can assure you that if we work together on this, you will soon feel much better.**		Emotional Talk_Reassurance / Optimism [High]<br>Partnering and activation_Paraphrase and interpretation [High]<br>Emotional Talk_Empathy [High]<br>Emotional Talk_Partnership [High]	<< empty >>	<< empty >>	Emotional Talk_Reassurance / Optimism [Low]<br>Partnering and activation_Paraphrase and interpretation [Low]<br>Emotional Talk_Empathy [Low]<br>Emotional Talk_Partnership [Low]
		Okay.<br>Great.		<< empty >>	<< empty >>	
	**Did you know** that the quality of the air plays a particularly important role in the subject of breathing? Air quality is naturally influenced by weather phenomena.		Emotional Talk_Partnership [High]	Above all, the quality of the air plays an important role in the subject of respiration. Air quality is of course also influenced by weather phenomena.		Emotional Talk_Partnership [Low]
		Okay.<br>I already knew that.		<< empty >>	<< empty >>	
	But it is the pollutants in the air that make breathing most difficult.			But it is the pollutants in the air that make breathing most difficult.		
		(A) That makes sense, but could you be a little more specific?			(A) That makes sense, but could you be a little more specific?	
		(B) I don't quite understand.			(B) I don't quite understand.	
	(A) Sure!			(A) Sure!		
	(B) No problem!			(B) No problem!		
	Air pollution has various causes and can occur everywhere.			Air pollution has various causes and can occur everywhere.		
	Exhaust fumes, for example, introduce many pollutants into our environment.			Exhaust fumes, for example, introduce many pollutants into our environment.		
		(A) Yeah, that makes sense.			(A) Yeah, that makes sense.	
		(B) I already knew that.			(B) I already knew that.	
	But that is not all.			But that is not all.		
	Tobacco smoke also represents a major pollution of the air we breathe.			Tobacco smoke also represents a major pollution of the air we breathe.		
	And even less obvious sources such as detergents, paints and varnishes or perfumes strongly influence the air we breathe.			And even less obvious sources such as detergents, paints and varnishes or perfumes strongly influence the air we breathe.		
		(A) That's frightening, but what can I do about it?			(A) That's frightening, but what can I do about it?	
		(B) I see. And now?			(B) I see. And now?	
	**What you can do for yoursel**f to relieve your discomfort is to keep your body fit.		Therapeutic regimen_closed-ended question [Low]	**You should keep your body fit and exercise as much as possible.**		Therapeutic regimen_closed-ended question [High]
Quiz	I have a quick quiz for you.			I have a quick quiz for you.		
		Okay, I'm curious.<br>Shoot.			Okay, I'm curious.<br>Shoot.	
	What do you think: Is the following statement right or wrong?			What do you think: Is the following statement right or wrong?		
	Cigarettes contain more than 4000 different chemical substances. Most of these substances are toxic and can cause respiratory diseases or cancer.			Cigarettes contain more than 4000 different chemical substances. Most of these substances are toxic and can cause respiratory diseases or cancer.		
		(A) Right.			(A) Right.	
		(B) Wrong.			(B) Wrong.	
	(A) Correct! Cigarettes are super harmful: they are the major risk factor for the development of COPD.			(A) Correct! Cigarettes are super harmful: they are the major risk factor for the development of COPD.		
	(B) **The statement was true - but don't worry, everyone makes mistakes.** Cigarettes are super harmful: they are the major risk factor for the development of COPD.		Emotional Talk_Reassurance / Optimism [High]	**(B) No, the statement is correct.** Cigarettes are super harmful: they are the major risk factor for the development of COPD.		Emotional Talk_Reassurance / Optimism [Low]
		Okay.<br>Got it.			Okay.<br>Got it.	
PatientEducation_ COPD2	**What is your attitude towards smoking?**		Partnering and activation_Asking for patient option [High]<br>Therapeutic regimen_closed-ended question [Low]	**Do you smoke?**		Partnering and activation_Asking for patient option [Low]<br>Therapeutic regimen_closed-ended question [High]
		**(A) I would like to stop - but it is difficult.**	Emotional Talk_Empathy [High]		(A) Yes.	Emotional Talk_Empathy [Low]
		**(B) Although I know it's harmful. But I don't really want to stop.**	Emotional Talk_Empathy [High]	<< empty >>	<< empty >>	Emotional Talk_Empathy [Low]
		(C) I do not smoke (anymore).			(B) I do not smoke (anymore).	
	**(A) I understand that, but it's the right attitude. We can do this together. I'm here for you.**		Emotional Talk_Reassurance / Optimism [High]<br>Emotional Talk_Partnership [High]	**(A) This is not good. You should really stop - smoking (but also passive smoke) is very harmful to your lungs and general health.**		Emotional Talk_Reassurance / Optimism [Low]<br>Emotional Talk_Partnership [Low]
	**(B) I understand that. Let's talk again later about why cigarettes are so important to you.**		Emotional Talk_Empathy [High]<br>Psychosocial Exchange about problems of daily living, issues about social relations, feelings, emotions [High]	**(B) Good, because smoking is very bad for you - even passive smoke.**		Emotional Talk_Empathy [Low]<br>Psychosocial Exchange about problems of daily living, issues about social relations, feelings, emotions [Low]
	**(C) Your lungs will thank you!**		Emotional Talk_Reassurance / Optimism [High]	<< empty >>	<< empty >>	Emotional Talk_Reassurance / Optimism [Low]
		Yeah, okay.			Yeah, okay.	
	In addition to tobacco smoke, however, there are numerous other pollutants in the air that pollute our airways and lungs.			In addition to tobacco smoke, however, there are numerous other pollutants in the air that pollute our airways and lungs.		
	If the airways are continuously attacked by pollutants, they become inflamed and swell.			If the airways are continuously attacked by pollutants, they become inflamed and swell.		
		Tell me more Robo.<br>This is interesting.			Tell me more Robo.<br>This is interesting.	
	The self-cleaning mechanism of the bronchi is then no longer effective.			The self-cleaning mechanism of the bronchi is then no longer effective.		
	Secretion accumulates in the bronchi and you have to cough more often.			Secretion accumulates in the bronchi and you have to cough more often.		
	If you produce secretion daily, you have chronic bronchitis.			If you produce secretion daily, you have chronic bronchitis.		
		(A) Exciting.			(A) Exciting.	
		(B) I already know all that.			(B) I already know all that.	
	(A) But there is more.			(A) But there is more.		
	(B) Do you also know the following?			(B) Do you also know the following?		
	Later the bronchial tubes become clogged and constricted, so that you get out of breath during exertion, later increasingly during everyday activities.			Later the bronchial tubes become clogged and constricted, so that you get out of breath during exertion, later increasingly during everyday activities.		
	Your breathing can become whistling.			Your breathing can become whistling.		
	With a lung function test, chronic obstruction, i.e. a permanent narrowing of the bronchi, can be detected.			With a lung function test, chronic obstruction, i.e. a permanent narrowing of the bronchi, can be detected.		
		(A) The problem looks familiar.			(A) The problem looks familiar.	
		(B) I didn't know that.			(B) I didn't know that.	

	That's why I'm here, **to help you the best I can.**		Emotional Talk_Empathy [High]	That's why I'm here.		Emotional Talk_Empathy [Low]
Quiz	But first a short quiz question again.			But first a short quiz question again.		
	What do you think the following statement is right or wrong?			What do you think the following statement is right or wrong?		
	Cigarette smoke slows down or even paralyses the self-cleaning mechanism of the airways.			Cigarette smoke slows down or even paralyses the self-cleaning mechanism of the airways.		
		(A) Right.			(A) Right.	
		(B) Wrong.			(B) Wrong.	
	(A) Right! Cigarettes are super harmful and one of the worst pollutants that can cause COPD.			(A) Right! Cigarettes are super harmful and one of the worst pollutants that can cause COPD.		
	(B) **The statement was true.** Cigarettes are super harmful and one of the worst pollutants that can cause COPD. **You will certainly answer the next quiz question correctly. I have complete confidence in you**.		Emotional Talk_Reassurance / Optimism [High]	(B) **Wrong!** Cigarettes are super harmful and one of the worst pollutants that can cause COPD.		Emotional Talk_Reassurance / Optimism [Low]
		Okay.<br>Got it.			Okay.<br>Got it.	
Exercise	In order to counteract the disease, it is important **that we** train your cardiovascular system with specific strengthening and endurance exercises. Physical training is the most important and effective non-drug measure you can take to influence the course of your disease yourself. **Did you already know that?**		Partnering and activation_Asking for patient option [High]	In order to counteract the disease, it is important to train the cardiovascular system through specific strengthening and endurance exercises. Physical training is the most important and effective non-drug measure you can take to influence the course of your disease yourself.		Partnering and activation_Asking for patient option [Low]
		This is news to me.<br>I already knew that.			This is news to me.<br>I already knew that.	
	**As a first exercise I suggest you to walk 10 steps without a break.**		Therapeutic regimen_closed-ended question [Low]	**As a first exercise you take 10 steps without a break.**		Therapeutic regimen_closed-ended question [High]
	<< empty >>	<< empty >>	Therapeutic regimen_closed-ended question [Low]	**You have to realize how important sports are to you.**	<< empty >>	Therapeutic regimen_closed-ended question [High]
	Are you ready?			Are you ready?		
