# Supplementary material for: Deliberative and Paternalistic Interaction Styles for Conversational Agents in Digital Health: Procedure and Validation Through a Web-Based Experiment
Source: J Med Internet Res. 2021 Jan 29;23(1):e22919. doi: 10.2196/22919 (PMC7880814; doi:10.2196/22919)
Supplement: Multimedia Appendix 4 [file jmir_v23i1e22919_app4.pdf]

Scenario Description German [original]:

Stellen Sie sich bitte folgendes Szenario vor und versuchen Sie, bei der Interaktion mit dem Chatbot so zu antworten, als ob Sie wirklich in dieser Situation wären.

Sie sind 65 Jahre alt und bereits in Rente. Sie leiden seit mehreren Jahren an einer chronischen Lungenkrankheit (COPD), die durch übermässigen und lang andauernden Zigarettenkonsum ausgelöst wurde. Aufgrund dieser Krankheit haben Sie Schwierigkeiten frei zu atmen, können eingeschränkt bis gar nicht mehr Sport betreiben, und seit dem letzten Krankheitsschub vor ein paar Monaten machen Ihnen vermehrt auch Tätigkeiten des Alltags (z.B. Einkaufen gehen) Probleme.

Sie sind nicht nur auf Medikamente angewiesen, die Ihnen den Alltag erleichtern sollen, sondern auch auf regelmässige Besuche beim Arzt. Dort wird Ihr aktueller Zustand bewertet sowie Anpassungen Ihrer Behandlung verordnet. Sie wissen, dass Ihre Krankheit unheilbar ist und nur mehr verlangsamt, nicht jedoch rückgängig gemacht werden kann. Ebenfalls wissen Sie, dass leichte physische Aktivität wichtig ist, um diese Verlangsamung zu unterstützen. Die verschriebenen Übungen zu machen fällt Ihnen in letzter Zeit jedoch immer schwerer – einerseits durch den Krankheitsschub, andererseits fehlt Ihnen mehr und mehr die Motivation.

Ihr Arzt verschreibt Ihnen nun bei Ihrem letzten Besuch einen sogenannten Chatbot namens "Robo", der spezifisch für COPD Patienten wie Sie entwickelt wurde. Ihr Arzt erklärt Ihnen, dass "Robo" zu jeder Zeit für Sie verfügbar ist und eine Ergänzung der Behandlung darstellt.

Sie sind sehr interessiert an so einer „digitalen Pille“ bzw. Ergänzung zu Ihren regelmässigen, jedoch weit auseinanderliegenden, Arztbesuchen und starten zuhause gleich mit der Interaktion….

Wenn Sie diesen Text sorgfältig gelesen haben, klicken Sie bitte auf den Weiter Button und beginnen Sie mit der Interaktion mit "Robo"!

Scenario Description English [translated by authors]:

Please imagine the following scenario and try to answer as if you were really in this situation when interacting with the chatbot.

You are 65 years old and already retired. You have been suffering for several years from a chronic lung disease (COPD), which is caused by excessive and prolonged cigarette consumption. Because of this disease you have difficulty breathing freely, you can do limited or no sport at all, and since the last episode a few months ago, you have had more problems with everyday activities (e.g. going shopping).

You are not only dependent on medication to make your everyday life easier, but also on regular visits to the doctor. There, your current condition will be assessed and adjustments to your treatment will be prescribed. You know that your illness is incurable and can only be slowed down, but not reversed. You also know that light physical activity is important to support this slowing down. However, you have been finding it increasingly difficult to do the prescribed exercises lately - on the one hand because of the relapse of your illness, on the other hand you are increasingly lacking motivation.

On your last visit, your doctor prescribed you a so-called chatbot called "Robo", which was developed specifically for COPD patients like you. Your doctor explained that "Robo" is available for you at any time and is a supplement to your treatment.

You are very interested in such a "digital pill" or supplement to your regular, but widely spaced, visits to your doctor and start interacting at home right away...

If you have read this text carefully, please click on the Next button and start interacting with "Robo"!
